# The *Salmonella* Effector SseK3 Targets Small Rab GTPases

**DOI:** 10.3389/fcimb.2020.00419

**Published:** 2020-08-19

**Authors:** Jiyao Gan, Nichollas E. Scott, Joshua P. M. Newson, Rachelia R. Wibawa, Tania Wong Fok Lung, Georgina L. Pollock, Garrett Z. Ng, Ian van Driel, Jaclyn S. Pearson, Elizabeth L. Hartland, Cristina Giogha

**Affiliations:** ^1^Centre for Innate Immunity and Infectious Diseases, Hudson Institute of Medical Research, Clayton, VIC, Australia; ^2^Department of Microbiology and Immunology, University of Melbourne at the Peter Doherty Institute for Infection and Immunity, Melbourne, VIC, Australia; ^3^Department of Biochemistry and Molecular Biology, Bio21 Molecular Science and Biotechnology Institute, The University of Melbourne, Parkville, VIC, Australia; ^4^Department of Molecular and Translational Science, Monash University, Clayton, VIC, Australia

**Keywords:** *Salmonella enterica*, Rab, glycosyltransferase, protein secretion, host-pathogen interaction

## Abstract

During infection, *Salmonella* species inject multiple type III secretion system (T3SS) effector proteins into host cells that mediate invasion and subsequent intracellular replication. At early stages of infection, *Salmonella* exploits key regulators of host intracellular vesicle transport, including the small GTPases Rab5 and Rab7, to subvert host endocytic vesicle trafficking and establish the *Salmonella-*containing vacuole (SCV). At later stages of intracellular replication, interactions of the SCV with Rab GTPases are less well defined. Here we report that Rab1, Rab5, and Rab11 are modified at later stages of *Salmonella* infection by SseK3, an arginine *N-*acetylglucosamine (GlcNAc) transferase effector translocated via the *Salmonella* pathogenicity island 2 (SPI-2) type III secretion system. SseK3 modified arginines at positions 74, 82, and 111 within Rab1 and this modification occurred independently of Rab1 nucleotide binding. SseK3 exhibited Golgi localization that was independent of its glycosyltransferase activity but Arg-GlcNAc transferase activity was required for inhibition of alkaline phosphatase secretion in transfected cells. While SseK3 had a modest effect on SEAP secretion during infection of HeLa229 cells, inhibition of IL-1 and GM-CSF cytokine secretion was only observed upon over-expression of SseK3 during infection of RAW264.7 cells. Our results suggest that, in addition to targeting death receptor signaling, SseK3 may contribute to *Salmonella* infection by interfering with the activity of key Rab GTPases.

## Introduction

*Salmonella enterica* Typhimurium (*S*. Typhimurium) is a common foodborne pathogen that imposes a significant financial burden on healthcare systems in both industrialized and developing countries (Majowicz et al., 2010). Upon ingestion of contaminated food or water, *Salmonellae* that survive the acidic gastric environment colonize the gut by invading both phagocytic and non-phagocytic cells (Clark et al., 1994; Smith, 2003; Geddes et al., 2007; Muller et al., 2012). *Salmonella* pathogenicity island 1 (SPI-1) and *Salmonella* pathogenicity island 2 (SPI-2) both encode type III secretion systems (T3SSs) that deliver virulence effector proteins into host cells that facilitate *Salmonella* pathogenesis. These two T3SSs are regulated in a spatio-temporal dependent manner, and are responsible for delivering two distinct cohorts of effectors into the host cell cytosol to hijack host physiology (LaRock et al., 2015). The SPI-1-encoded T3SS is activated initially, inducing inflammation and host cytoskeletal rearrangements to promote invasion of the bacteria into epithelial cells, while cells such as macrophages internalize *Salmonella* by phagocytosis. Following internalization, the SPI-2-encoded T3SS is activated to establish a membrane-bound replicative niche termed the *Salmonella-*containing vacuole (SCV). The SCV subverts the canonical endolysosomal pathway to facilitate intracellular bacterial survival and replication (Ramsden et al., 2007; Jennings et al., 2017).

SseK1, SseK2, and SseK3 are three SPI-2 translocated effectors which show high amino acid sequence similarity (84, 83, and 80% respectively) to the T3SS effector NleB1 from enteropathogenic *Escherichia coli* (EPEC) (Kujat Choy et al., 2004; Brown et al., 2011; Li et al., 2013; Pearson et al., 2013). NleB1 is a novel glycosyltransferase that blocks apoptotic cell death during EPEC infection by modifying conserved arginine residues with *N-*acetylglucosamine (GlcNAc) within death domain containing proteins, including FADD and TRADD (Li et al., 2013; Pearson et al., 2013; Scott et al., 2017). The post-translational modifications are termed Arg-GlcNAcylation and are only mediated by members of the NleB1 effector protein homolog family including the SseKs (El Qaidi et al., 2017; Gunster et al., 2017; Esposito et al., 2018; Park et al., 2018; Newson et al., 2019). Similar to NleB1, expression of SseK1 or SseK3, but not SseK2, suppresses NF-κB activation during infection, although these effectors exhibit different Arg-GlcNAcylation profiles on infected host cells (Gunster et al., 2017; Newson et al., 2019). Our group recently showed that overexpression of SseK1 and SseK3 resulted in broadened substrate specificity, suggesting that authentic host targets of these effectors need to be identified under native expression conditions during infection (Newson et al., 2019). When expressed at endogenous levels during *Salmonella* infection, SseK1 preferentially modifies the death domain of TRADD, whereas SseK3 targets death domains in the death receptors, TRAILR and TNFR1 (Newson et al., 2019). Interestingly, both SseK2 and SseK3 localize to the Golgi during infection; while SseK1 localizes to the cytosol (Gunster et al., 2017). Such observations suggest SseK2 and SseK3 may have uncharacterized Golgi-localized targets.

In this study, we sought to identify Golgi-associated proteins that are modified by SseK3 during *S*. Typhimurium infection. Using a mass spectrometry-based approach to examine membrane-enriched fractions of infected macrophages, we identified several Rab GTPases as targets of SseK3, including Rab1, Rab5, and Rab11. Notably, we found SseK3 modified arginine residues in the switch II region of Rab1, and that ectopic expression of either SseK2 or SseK3, but not SseK1, blocked host protein secretion. However, only SseK3 appeared to have the capacity to block host protein secretion during *Salmonella* infection. Collectively, these results suggest that SseK3 may contribute to *Salmonella* infection by GlcNAcylating selected Rab GTPases.

## Materials and Methods

### Bacterial Strains and Growth Conditions

The bacterial strains used in this study are listed in Table 1. All bacteria were grown in Luria-Bertani (LB) broth with shaking at 200 rpm in 37°C incubator in the presence of streptomycin (50 μg/ml), kanamycin (100 μg/ml), or ampicillin (100 μg/ml) when necessary.

**Table 1:** List of bacterial strains used in this study.

**Strain**	**Characteristics**	**Source/References**
SL1344	*S. enterica* serovar Typhimurium strain SL1344	Hoiseth and Stocker, 1981
Δ*sseK123*	SL1344 Δ*sseK1ΔsseK2ΔsseK3*	Brown et al., 2011
Δ*sseK23*	SL1344 Δ*sseK2ΔsseK3*	Brown et al., 2011
Δ*sseK12*	SL1344 Δ*sseK1ΔsseK2*	Kujat Choy et al., 2004
Δ*sseK23* (pSseK2)	SL1344 Δ*sseK2ΔsseK3* supplemented with pTrc99A-SseK2 plasmid	This study
Δ*sseK23* (pSseK3)	SL1344 Δ*sseK2ΔsseK3* supplemented with pTrc99A-SseK3 plasmid	This study
Δ*ssaR*	SL1344 Δ*ssaR* (SPI-2 mutant)	Kupz et al., 2012

### DNA Cloning and Purification

The plasmids and primers used in this study are listed in Tables 2, 3 respectively. All plasmid extractions were performed using QIAGEN QIAprep Miniprep Kit (Qiagen, Valencia, CA). DNA restriction digests were applied according to manufacturer's instructions (New England Biolabs, Ipswich, MA). PCR products, restriction digest products and DNA from agarose gels were purified using the Wizard SV Gel and PCR Clean-Up system (Promega, Madison, WI). Digested inserts and plasmids were ligated with a 6:1 molar ratio at 4°C overnight using T4 DNA Ligase system in accordance with manufacturer's instructions (Promega, Madison, WI). The pF_TRE-SEAP ligation product was transformed into DH5α cells and other resultant ligation products were transformed into XL1-Blue cells. The pF_TRE-SEAP plasmid was sequenced with pFTRE-F/R and other plasmids were extracted and sequenced with sequencing primer pair p3xFlag-Myc-CMV-24F/p3xFlag-Myc-CMV-24R.

**Table 2:** List of plasmids used in this study.

**Plasmid**	**Characteristics**	**Source/References**
pEGFP-C2	Mammalian expression vector expressing EGFP fused to the N terminus of the encoding protein, Kan^R^	Clontech
pEGFP-C2-SseK1	*sseK1* from *S*. Typhimurium SL1344 in pEGFP-C2, Kan^R^	Newson et al., 2019
pEGFP-C2-SseK2	*sseK2* from *S*. Typhimurium SL1344 in pEGFP-C2, Kan^R^	Newson et al., 2019
pEGFP-C2-SseK3	*sseK3* from *S*. Typhimurium SL1344 in pEGFP-C2, Kan^R^	Newson et al., 2019
pEGFP-C2-SseK1_DXD_	*sseK1* from *S*. Typhimurium SL1344 in pEGFP-C2, with catalytic motif DxD(229-231) mutated to AAA, Kan^R^	This study
pEGFP-C2-SseK2_DXD_	*sseK2* from *S*. Typhimurium SL1344 in pEGFP-C2, with catalytic motif DxD(293-241) mutated to AAA, Kan^R^	This study
pEGFP-C2-SseK3_DXD_	*sseK3* from *S*. Typhimurium SL1344 in pEGFP-C2, with catalytic motif DxD(226-228) mutated to AAA, Kan^R^	This study
p3xFlag-*Myc-*CMV-24	Mammalian expression vector with Met-3xFlag tagged at N-terminal and *c-myc* tagged at C-terminal, Amp^R^	Signa-Aldrich
p3xFlag-Rab1a	Human Rab1a in p3xFlag-*Myc-*CMV-24, Amp^R^	This study
p3xFlag-Rab1a_R74A_	Human Rab1a with Arg74 mutated to Ala74 in p3xFlag-*Myc-*CMV-24, Amp^R^	This study
p3xFlag-Rab1a_R82A_	Human Rab1a with Arg82 mutated to Ala82 in p3xFlag-*Myc-*CMV-24, Amp^R^	This study
p3xFlag-Rab1a_R111A_	Human Rab1a with Arg111 mutated to Ala111 in p3xFlag-*Myc-*CMV-24, Amp^R^	This study
p3xFlag-Rab1a_R74AR82A_	Human Rab1a with Arg74 and Arg82 mutated to Ala74 and Ala82, respectively, in p3xFlag-*Myc-*CMV-24, Amp^R^	This study
p3xFlag-Rab1a_R74AR111A_	Human Rab1a with Arg74 and Arg111 mutated to Ala74 and Ala111, respectively, in p3xFlag-*Myc-*CMV-24, Amp^R^	This study
p3xFlag-Rab1a_R82AR111A_	Human Rab1a with Arg82 and Arg111 mutated to Ala82 and Ala111, respectively, in p3xFlag-*Myc-*CMV-24, Amp^R^	This study
p3xFlag-Rab1a_R74AR82AR111A_	Human Rab1a with Arg74, Arg82, and Arg111 mutated to Ala74, Ala82 and Ala111in p3xFlag-*Myc-*CMV-24, Amp^R^	This study
p3xFlag-Rab5a	Human Rab5a in p3xFlag-*Myc-*CMV-24, Amp^R^	This study
p3xFlag-Rab5b	Human Rab5b in p3xFlag-*Myc-*CMV-24, Amp^R^	This study
p3xFlag-Rab5c	Human Rab5c in p3xFlag-*Myc-*CMV-24, Amp^R^	This study
p3xFlag-Rab11b	Human Rab11b in p3xFlag-*Myc-*CMV-24, Amp^R^	This study
pTrc99A-SseK2	*sseK2* from *S*. Typhimurium SL1344 in pTrc99A, Amp^R^	Newson et al., 2019
pTrc99A-SseK3	*sseK3* from *S*. Typhimurium SL1344 in pTrc99A, Amp^R^	Newson et al., 2019
pSEAP	Secreted embryonic alkaline phosphatase in a mammalian expression vector	Kagan et al., 2004
p3xFlag-AnkX	*ankX* from *L.pneumophila* in p3xFlag-*Myc-*CMV-24, Amp^R^	This study
pF_TRE3G_PGK puro	Lentiviral transduction vector, AMP^R^	Yamamoto et al., 2012
pF_TRE-SEAP	*seaP* from pSEAP in pF_TRE3G_PGK puro, Amp^R^	This study
pCMV-VSV-G	Mammalian expression vector expressing VSV-G glycoprotein, AMP^R^	Stewart et al., 2003
pCMVΔR8.2	Mammalian expression vector expressing HIV-1 Gag/Pol, Tat, and Rev, AMP^R^	Stewart et al., 2003

**Table 3:** List of primers used in this study.

**Name**	**Primer sequences 5'-3'**
Rab1a_F_	CGCGATATCGATGTCCAGCATGAATCCCG
Rab1a_R_	CGCGGATCCTTAGCAGCAACCTCCACCTG
Rab1a_R74A−F_	AGGCCAGGAAAGATTTGCAACAATCACCTCCAGTT
Rab1a_R74A−R_	AACTGGAGGTGATTGTTGCAAATCTTTCCTGGCCT
Rab1a_R82A−F_	ACCTCCAGTTATTACGCAGGAGCCCATGGCATCA
Rab1a_R82A−R_	TGATGCCATGGGCTCCTGCGTAATAACTGGAGGT
Rab1a_R111A−F_	GGCTGCAGGAAATAGATGCATATGCCAGTGAAAATGT
Rab1a_R111A−R_	ACATTTTCACTGGCATATGCATCTATTTCCTGCAGCC
Rab5a_F_	CCCAAGCTTATGGCTAGTCGAGGCGCAA
Rab5a_R_	CGCGGATCCTTAGTTACTACAACACTGATTCCTGGTT
Rab5b_F_	CCCAAGCTTATGACTAGCAGAAGCACAGCTAGG
Rab5b_R_	CGCGGATCCTCAGTTGCTACAACACTGGCTCTT
Rab5c_F_	CGCGATATCGATGGCGGGTCGGGGAGG
Rab5c_R_	CGCGGATCCTCAGTTGCTGCAGCACTGGCT
Rab11b_F_	CCCAAGCTTATGGGGACCCGGGACGAC
Rab11b_R_	CGCGGATCCTCACAGGTTCTGGCAGCACTGC
p3xFlag-Myc-CMV-24F	AATGTCGTAATAACCCCGCCCCGTTGACGC
p3xFlag-Myc-CMV-24R	TATTAGGACAAGGCTGGTGGGCAC
AnkX_F_	AAAGTCGACATGCCAAATCTACCTGG
AnkX_R_	TTTGGATCCTTACCATTTTAATTTCAAGG
SseK1_AAA−F_	GGTGTATATATCTTGCTGCTGCTATGATTATCACGGAAAAACTGG
SseK1_AAA−R_	CCAGTTTTTCCGTGATAATCATAGCAGCAGCAAGATATATACACC
SseK2_AAA−F_	GTGGGTGCATATATCTTGCTGCAGCTATGTTACTTACTGATAAAC
SseK2_AAA−R_	GTTTATCAGTAAGTAACATAGCTGCAGCAAGATATATGCACCCAC
SseK3_AAA−F_	CTGGAGGTGGCTGCATATATCTTGCTGCTGCTATGTTACTTACAG
SseK3_AAA−R_	CTGTAAGTAACATAGCAGCAGCAAGATATATGCAGCCACCTCCAG
SEAP_F_	CGCTGATCAATGCTGCTGCTGCTGCTGCTGCTG
SEAP_R_	CTAGCTAGCTCATGTCTGCTCGAAGCGG
pFTRE-F	GTGTACGGTGGGCGCC
pFTRE-R	GTTGGCGCCTACCGGTG

The p3xFlag-Rab1a vector was constructed by amplifying *RAB1A* from human cDNA using primer pair Rab1a_F/_Rab1a_R_, which was then double digested with *EcoRV* and *BamHI* and ligated into p3xFlag-*Myc*-CMV-24 plasmid that had been digested with same restriction enzymes to generate an N-terminal 3xFlag fusion to Rab1a. p3xFlag-Rab5a and p3xFlag-Rab5b were constructed in a similar manner by amplifying *RAB5A* or *RAB5B* from human cDNA using primer pair Rab5a_F/_Rab5a_R_ or Rab5b_F/_Rab5b_R_ respectively, which were then double digested with *HindIII* and *BamHI* and ligated into p3xFlag-*Myc*-CMV-24. p3xFlag-Rab5c was constructed by amplifying *RAB5C* from human cDNA using primer pair Rab5c_F/_Rab5c_R_, which was then double digested with *EcoRV* and *BamHI* and ligated into p3xFlag-*Myc*-CMV-24. p3xFlag-Rab11b was constructed by amplifying *RAB11B* from human cDNA using primer pair Rab11b_F/_Rab11b_R_, which was then double digested with *HindIII* and *BamHI* and ligated into p3xFlag-*Myc*-CMV-24. p3xFlag-AnkX was constructed by amplifying *ankX* from *Legionella pneumophila* Philadelphia 1 genomic DNA using primer pair AnkX_F/R_, double digested with *SalI* and *BamHI* and ligated into p3xFlag-*Myc*-CMV-24 plasmid that has been digested with same restriction enzymes to generate an N-terminal 3xFlag fusion to AnkX. The pF_TRE-SEAP was constructed by amplifying *SEAP* from pSEAP vector using primer pair SEAP_F_/SEAP_R_, which was subsequently double digested with *BclI* and *NheI* and ligated into pF_TRE3G_PGK puro that has been digested with same restriction enzymes.

### Site-Directed Mutagenesis

All site-directed mutagenesis was performed using QuikChange II Site-Directed Mutagenesis Kit according to manufacturer's protocol. p3xFlag-Rab1a_R74A_ was generated with primer pair Rab1a_R74A−F_/Rab1a_R74A−R_ using p3xFlag-Rab1a vector as a template. Rab1a_R82A_ was generated with primer pair Rab1a_R82A−F_/Rab1a_R82A−R_ using p3xFlag-Rab1a vector as a template. Rab1a_R111A_ was generated with primer pair Rab1aR_111A−F_/Rab1aR_111A−R_ using p3xFlag-Rab1a vector as a template. Rab1a_R74AR82A_ and Rab1a_R74AR111A_ were generated with primer pair Rab1a_R82A−F_/Rab1a_R82A−R_ and Rab1a_R111A−F_/Rab1a_R111A−R_ respectively using p3xFlag-Rab1a_R74A_ vector as a template. Rab1a_R82AR111A_ was generated with primer pair Rab1a_R111A−F_/Rab1a_R111A−R_ using p3xFlag-Rab1a_R82A_ vector as a template. Rab1a_R74AR82AR111A_ was generated with primer pair Rab1a_R111A−F_/Rab1a_R111A−R_ using p3xFlag-Rab1a_R74AR82A_ vector as a template. The sequences of resulting plasmids were confirmed by sequencing using primer pair p3xFlag-Myc-CMV-24F/p3xFlag-Myc-CMV-24R. The pEGFP-C2-SseK1_DXD_, pEGFP-C2-SseK2_DXD_ and pEGFP-C2-SseK3_DXD_ vectors were generated using primer pairs SseK1_AAA−F/R_, SseK2_AAA−F/R_ and SseK3_AAA−F/R_ respectively_._ All resultant plasmids following PCR reactions were digested with *DpnI* at 37°C overnight and then transformed into XL1-Blue competent cells.

### Arg-GlcNAcylation Pull Downs on Insoluble Fractions of *Salmonella* Infected RAW264.7 Cells

RAW264.7 cells were seeded to 24 well plates at a concentration of 3 × 10^5^ cells per well 1 day before infection. 10 ml LB broths containing appropriate antibiotic were inoculated with *Salmonella* strains and incubated at 37°C overnight with shaking at 180 rpm. On the day of infection, the OD600 readings of the overnight culture were read and used to estimate bacterial counts. Cells were then infected at a multiplicity of infection (MOI) of 10. 24 well plates were centrifuged at 1500 rpm for 5 min at room temperature to promote and synchronize infection. Infected cells were incubated at 37°C, 5% CO_2_ for 1 h. Culture media was replaced with media containing 100 μg/ml gentamicin (Pharmacia, Washington, USA), and cells were incubated at 37°C, 5% CO_2_ for a further 1 h. Culture media was replaced with media containing 10 μg/ml gentamicin, and cells were incubated at 37°C, 5% CO_2_ for a further 18 h.

Infected cells were washed three times in ice-cold PBS, then collected by scraping in PBS containing cOmplete™ EDTA-free protease inhibitor cocktail (Roche), on a bed of ice. Cells were centrifuged at 4000 rpm for 5 min at 4°C, and the supernatant was carefully removed via aspiration using a vacuum pump. Cells were resuspended in 1 ml of ice-cold lysis buffer (20 mM HEPES (pH 7.5), 100 mM KCl, 2.5 mM MgCl_2_, 100 mM sucrose, 10% PhosSTOP™ (Sigma-Aldrich), and cOmplete™ EDTA-free protease inhibitor cocktail (Roche). Cells were mixed thoroughly by pipette and incubated on ice for 10 min. Cell lysates were centrifuged at 13000 rpm for 5 min at 4°C, and the supernatant was removed by vacuum-aided aspiration as previously. Pelleted material was resuspended in lysis buffer containing 10% digitonin, mixed thoroughly by pipette and incubated on ice for 1 h with intermittent vortexing. Cell lysates were centrifuged at 13000 rpm for 5 min at 4°C, and the supernatant was collected as the digitonin-soluble membrane fraction, while the remaining pellet was collected as the digitonin-insoluble fraction.

Insoluble membrane pellets were resuspended in 8M urea in 100 mM ammonium bicarbonate and protein concentration determined using a BCA assay. 2.5 mg of protein from each sample type, Δ*sseK12* and Δ*sseK123*, were reduced with 10 mM dithiothreitol for 1 h then alkylated with 50 mM chloroacetamide for a further 1 h in the dark. Samples were digested with Lys-C (1/200 w/w) for 3 h at RT, samples diluted to <2M urea and digested with trypsin (1/100 w/w) overnight at RT. Digested samples were acidified to a final concentration of 0.5% formic acid and desalted with 50 mg tC18 SEP-PAK (Waters corporation, Milford, USA) according to the manufacturer's instructions. Briefly, tC18 SEP-PAKs were conditioned with buffer B (80% acetonitrile, 0.1% formic acid), washed with 10 volumes of Buffer A^*^ (0.1% trifluoroacetic acid, 2% acetonitrile), sample loaded, column washed with 10 volumes of Buffer A^*^ and bound peptides eluted with buffer B then dried.

Peptide affinity purification was accomplished according to the protocol of Scott et al. (2017) for peptide based Arg-GlcNAc enrichment. Briefly, aliquots of 100 μl of Protein A/G plus Agarose beads (Santa Cruz, Santa Cruz CA) were washed three times with 1 ml of immunoprecipitation buffer (IAP, 10 mM Na_2_HPO_4_, 50 mM NaCl, 50 mM MOPS, pH 7.2) and tumbled overnight with 10 μg of anti-Arg-GlcNAc antibody (ab195033, Abcam) at 4°C. Coupled anti-Arg-GlcNAc beads were then washed three times with 1 ml of 100 mM sodium borate (pH 9) to remove non-bound proteins and cross-linked for 30 min by gently rotating with 20 mM Dimethyl Pimelimidate (Thermo Fisher Scientific) in 100 mM HEPES, pH 8.0. Cross-linking was quenched by washing beads with 200 mM ethanolamine, pH 8.0, three times then rotating the beads in an additional 1 ml 200 mM ethanolamine, pH 8.0 for 2 h at 4°C. Beads were washed three times with IAP buffer and used immediately. Purified peptides were resuspended in 1 ml IAP buffer and the pH checked to ensure compatibility with affinity conditions (~pH7.2). Peptide lysates were then added to the prepared cross-linked anti-Arg-GlcNAc antibody beads and rotated for 3 h at 4°C. Upon completion of the incubation, antibody beads were spun down at 3000 G for 2 min at 4°C and the unbound peptide lysates collected. Antibody beads were then washed six times with 1 ml of ice-cold IAP buffer and Arg-GlcNAc peptides eluted using two rounds of acid elution. For each elution round, 100 μl of 0.2% trifluoroacetic acid was added and antibody beads allowed to stand at room temperature with gentle shaking every minute for 10 min. Peptide supernatants were collected and desalted using C18 stage tips (Rappsilber et al., 2007) before analysis by LC-MS.

### Identification of Arginine-Glycosylated Affinity Enriched Peptides and Flag-Tagged Proteins Using Reversed Phase LC-MS

Purified peptides prepared were re-suspend in Buffer A^*^ and separated using a two-column chromatography set up composed of a PepMap100 C18 20 mm × 75 μm trap and a PepMap C18 500 mm × 75 μm analytical column (Thermo Fisher Scientific). Samples were concentrated onto the trap column at 5 μl/min for 5 min and infused into an Orbitrap Fusion™ Lumos™ Tribrid™ Mass Spectrometer (Thermo Fisher Scientific) at 300 nl/minute via the analytical column using a Dionex Ultimate 3000 UPLC (Thermo Fisher Scientific). 125 min gradients were run altering the buffer composition from 1% buffer B to 28% B over 90 min, then from 28% B to 40% B over 10 min, then from 40% B to 100% B over 2 min, the composition was held at 100% B for 3 min, and then dropped to 3% B over 5 min and held at 3% B for another 15 min. The Lumos™ Mass Spectrometer was operated in a data-dependent mode automatically switching between the acquisition of a single Orbitrap MS scan (120,000 resolution) every 3 s and Orbitrap HCD for each selected precursor (maximum fill time 100 ms, AGC 2^*^10^5^ with a resolution of 30,000 for Orbitrap MS-MS scans).

### Mass Spectrometry Data Analysis

Identification of proteins and Arg-glycosylated peptides was accomplished using MaxQuant (v1.5.3.30) (Cox and Mann, 2008). Searches were performed against the Mouse (Uniprot proteome id UP000000589—Mus musculus, downloaded 18-05-2016, 50306 entries) and *Salmonella* Typhimurium SL1344 (Uniprot proteome id UP000008962- *Salmonella* Typhimurium SL1344, downloaded 18-05-2016, 4,657 entries) proteomes with carbamidomethylation of cysteine set as a fixed modification. Searches were performed with trypsin cleavage specificity allowing 2 mis-cleavage events and the variable modifications of oxidation of methionine, *N*-Acetylhexosamine addition to arginine (Arg-GlcNAc) and acetylation of protein N-termini. The precursor mass tolerance was set to 20 parts-per-million (ppm) for the first search and 10 ppm for the main search, with a maximum false discovery rate (FDR) of 1.0% set for protein and peptide identifications. To enhance the identification of peptides between samples the Match Between Runs option was enabled with a precursor match window set to 2 min and an alignment window of 10 min. For label-free quantitation, the MaxLFQ option within Maxquant (Cox et al., 2014) was enabled in addition to the re-quantification module. The resulting protein group output was processed within the Perseus (v1.4.0.6) (Tyanova et al., 2016) analysis environment to remove reverse matches and common protein contaminates prior. For LFQ comparisons missing values were imputed using Perseus. Enrichment analysis of Arg-GlcNAcylated targets was undertaken using a Fisher exact test within Perseus. Data was exported into the R framework for visualization using ggplot2. The mass spectrometry proteomics data have been deposited to the ProteomeXchange Consortium via the PRIDE partner repository with the dataset identifier PXD015082.

### Construction of Stable Cell Lines Expressing SEAP in HeLa229 Cells

HEK293T cells were seeded in a 10 cm plate and grown to 60% confluency before being transfected with 1.2 μg pF_TRE-SEAP, 2.0 μg pCMVΔR8.2 and 0.8 μg pCMV-VSV-G. Transfected cells were kept in a tissue culture incubator under 5% CO_2_ at 37°C for 24 h before the tissue culture media was replaced. The supernatant containing packaged virus was collected 48 h post transfection and then filtered through a 0.45 μm filter. HeLa229 cells were seeded in a 6 well plate and grown to 50% confluency before infection. 5 μg/ml Polybrene (Sigma) was added to virus containing media. DMEM GlutaMAX (Gibco) supplemented with 10% (v/v) Fetal Bovine Serum (FBS, RBS Thermo scientific) was mixed with virus containing media in a 1:1 ratio before being added to HeLa229 cells which were then centrifuged at 1,500 rpm for 30 min at room temperature. Infected cells were incubated for 48 h and then the tissue culture medium was replaced with DMEM GlutaMAX (Gibco) supplemented with 10% (v/v) Fetal Bovine Serum (FBS, RBS Thermo scientific) containing 5 μg/ml puromycin (Sigma). Infected cells were selected with 5 μg/ml puromycin for 3 passages before use.

### Mammalian Cell Culture

HEK293T, RAW264.7, HeLa229 and HeLa229 SEAP cells were cultured in T75 cm^2^ or T25 cm^2^ tissue culture flasks (Corning) in DMEM GlutaMAX (Gibco) supplemented with 10% (v/v) Fetal Bovine Serum (FBS, RBS Thermo scientific) in a tissue culture incubator under 5% CO_2_ at 37°C. All cell lines were kept and passaged to maximum of 40 times. HeLa229 cells, HeLa229 SEAP cells and HEK293T cells were split when cells were grown to 80 to 90% confluency. When splitting cells, cells were washed two times with 1 × PBS followed with 0.4 ml for T25 cm^2^ flask or 1.0 ml for T75 cm^2^ flask of 0.05% Trypsin-EDTA solution (Gibco, Life Technologies) treatment for 1 min at 37°C. 0.05% Trypsin-EDTA solution treated cells were resuspended with 10 ml of DMEM media supplemented with 10% FBS. RAW264.7 cells were washed two times with 1 x PBS and detached from tissue culture flasks with cell scrapers and diluted in fresh DMEM media supplemented with 10% FBS.

### Immunoblotting

Cells were collected and placed on ice, 1xKal-B cell lysis buffer supplemented with protein inhibitors [50 mM Tris-HCl pH 7.4, 1 mM EDTA, 150 mM NaCl, 1% Triton X-100, 10 mM NaF, 1 mM PMSF, 2 mM Na_3_VO_4_, and 1 x EDTA-free Complete protease inhibitor mixture (Roche)] was used to lyse harvested cells. Collected samples were placed on ice for 30 min, with pipetting up and down every 10 min to complete lysis cells. Cell lysates were pelleted at 13,000 rpm for 10 min. 4 × Bolt® LDS sample buffer (Life Technologies) and DTT (Astral scientific) at a final concentration of 50 mM were added to supernatants of samples and boiled at 70°C for 10 min. Boiled samples were loaded on Bolt® 4–12% Bis-Tris Plus gels (Life Technologies) and electrophoresis was performed according to manufacturer's instructions. Proteins were then transferred to nitrocellulose membranes using iBlot® nitrocellulose transfer stacks (Life Technologies) following manufacturer's protocol. Transferred nitrocellulose membranes were then blocked with 5% (w/v) skim milk in 1 x TBST buffer (20 mM Tris, 50 mM NaCl, 0.1% (v/v) Tween-20, pH 8.0) at room temperature with shaking at 60 rpm for 1 h. Blocked membranes were washed with 1 x TBST buffer 3 times, 5 min for each wash, before being probed with primary antibodies as required at 4°C, shaking at 60 rpm overnight. Primary antibodies are used as follows: mouse monoclonal anti-GFP (1:2000 dilution) (Roche, Basel, Switzerland), mouse monoclonal anti-Flag M2-HRP (1:2000 dilution, Sigma-Aldrich, St Louis, MO), or mouse monoclonal anti-β-actin (1:5000 dilution, Sigma-Adrich, St Louis, MO), or rabbit monoclonal anti-ArgGlcNAc (1:2000 dilution, Abcam, Cambridge, UK). All primary antibodies were diluted in 5% bovine serum albumin (Sigma-Aldrich) in 1 x TBST buffer. Membranes were then washed 3 times, for 5 min each, in 1 × TBST at room temperature with shaking at 60 rpm. Secondary antibodies, if required, were then incubated on membranes at room temperature with shaking at 60 rpm for 1 h. Secondary antibodies used in this study were horseradish peroxidase (HRP) conjugated anti-mouse (PerkinElmer), or HRP conjugated anti-rabbit (Bio-Rad). All secondary antibodies were diluted at 1:3000 with 5% bovine serum albumin (Sigma-Aldrich) in 1 × TBST buffer. Probed membranes were washed 7 times, for 5 min each, with 1 × TBST at room temperature with shaking at 60 rpm before being developed using ECL Prime Western blotting reagent (Amersham Bioscience) according to manufacturer's instructions. Immunoblots were developed using the Amersham Imager 680 blot and gel imager (GE Healthcare).

### Transfection of HEK293T Cells

HEK293T cells were seeded in 24 well plates (Greiner Bio-One) at a concentration of 1 × 10^5^ cells per well on a coverslip for immunofluorescence experiments or at 2 × 10^5^ cells per well for secreted embryonic alkaline phosphatase assays. For immunoprecipitation, HEK293T cells were seeded in 10 cm cell culture dishes (Greiner Bio-One) at 4 × 10^6^ cells per dish. Cells were seeded 1 day before transfection. On the day of transfection, FuGENE 6 Transfection Reagent (Roche) was added into Opti-MEM® I (1x) in GlutaMAX(TM)-I (Gibco, Life Technologies) and incubated for 5 min at room temperature before mixed with relevant plasmids according to manufacturer's instructions. Transfection mixtures were incubated for 20 min before added to seeded cells. Transfected cells were placed in 37°C incubator with 5% CO_2_ for 18 h incubation before harvesting for other experiments.

### Immunoprecipitation of Flag Tagged Fusion Proteins

Harvested HEK293T cells were collected using 1 × KalB buffer supplemented with protein inhibitors as described above. Samples were placed on ice for 30 min, with pipetting up and down every 10 min, for complete cell lysis. Cell debris was pelleted at 13,000 rpm for 10 min at 4°C. Anti-Flag® M2 Magnetic Beads (Sigma-Aldrich) were washed 3 times, for 5 min each with 1 × Kal-B buffer before being loaded with cell lysates. Cell lysates were incubated with the beads with rotation at 4°C overnight. Flag-tagged proteins were eluted with 80 μl of 150 μg/ml Flag peptide (Sigma-Aldrich) with rotation at 4°C for 30 min. Eluted samples were processed for immunoblotting as described above.

### Secreted Embryonic Alkaline Phosphatase Assay

For transfection on HEK293T cells, pSEAP vector was used to co-transfect HEK293T cells with vectors expressing AnkX, SseKs or their catalytic mutants following the transfection method described above. 16 h post transfection or infection, 200 μl DMEM GlutaMAX (Gibco) media was added to each well to replace old media and the 24 well plate was put back at 37°C with 5% CO_2_ for a further 8 h incubation. 24 h post transfection or infection, supernatants and cells lysates were collected for processing using Phospha-Light^TM^ SEAP Reporter Gene Assay System (ThermoFisher Scientific) following manufacturer's instructions. Processed samples were plated on a 96-well white flat bottom plate and read via CLARIOstar Plus (BMG LABTECH).

### Infection of Mammalian Cell Lines

RAW264.7 cells were seeded in 24 well plates at a concentration of 1 × 10^5^ cells per well for cytometric bead array assay 1 day before infection. HeLa229 SEAP cells were seeded in 24 well plates at a concentration of 1 x 10^5^ cells per well 1 day before experiment. *Salmonella* strains were inoculated in LB broth with relevant antibiotics 1 day before infection and grown at 37°C with shaking at 200 rpm overnight. On the day of infection, *Salmonella* strains were sub-cultured at a ratio of 1:100 in fresh LB broth with relevant antibiotics and grown at 37°C with shaking at 200 rpm for 3.5 h. OD_600_ of different sub-cultures were recorded using a spectrophotometer (SPECTRONIC^TM^ 200, Thermo Scientific^TM^) to estimate bacterial density. Sub-cultured *Salmonella* was diluted 10-fold in DMEM GlutaMAX (Gibco) media and added into seeded cells at a multiplicity of infection (MOI) of 10. The plates were centrifuged at 1,500 rpm for 5 min to synchronize the infection and incubated at 37°C with 5% CO_2_ for 30 min to allow for invasion. The cells were then washed twice with 1 x PBS and incubated with fresh DMEM GlutaMAX (Gibco) media supplemented with 100 μg/ml gentamicin and further incubated at 37°C with 5% CO_2_ for 1 h. After 1 h incubation, the cells were washed twice with 1 x PBS and incubated with fresh DMEM GlutaMAX (Gibco) media supplemented with 10 μg/ml gentamicin, and where necessary, a final concentration of 1 mM IPTG was added into the media. Infected cells were incubated at 37°C with 5% CO_2_ for various timepoints. For infection on HeLa229 SEAP cells, the media was replaced and 100 ng/ml doxycycline (Sigma) was used to induce SEAP expression where necessary; for cytometric bead array assays, the media was replaced on infected cells at 16 h post infection, and supernatants were collected at 20 or 24 h post infection when required.

### Intracellular Replication of *Salmonella* Strains in HeLa229 SEAP and RAW264.7 Cells

Infection of HeLa229-SEAP and RAW264.7 cells with derivatives of *Salmonella* SL1344 was carried out as described above. Infected cells were collected for enumeration of bacteria at 2 and 24 h post infection. Infected cells were washed twice in PBS before being lysed for 5 min in 250 μl 0.1% Triton X-100 before been scraped and collected. Cell lysates were serially diluted in PBS, and then plated on LB agar plates containing 50 μg/ml streptomycin. Plated LB agar plates were incubated at 37°C overnight. *Salmonella* colonies were enumerated, and the colony-forming units (CFU) were calculated for each time point. Fold replication for each *Salmonella* strain was determined by dividing CFU at 24 h post infection by CFU at 2 h post infection.

### Immunofluorescence Microscopy

Cells grown on coverslips were fixed with 4% paraformaldehyde in 1 × PBS for 10 min at room temperature at required time points following transfection. Cells were permeabilized using 0.2% Triton X-100 in PBS for 3 min, and then blocked with 3% BSA in 1 × PBS for 1 h. Primary antibodies were diluted at 1:200 in 3% BSA in 1 × PBS and cells were stained for 1 h at room temperature. Primary antibodies used for immunofluorescence were: monoclonal anti-Flag M2 (Sigma-Aldrich), polyclonal anti-Golgin 97 (Abcam, ab84830). The cells were then washed 3 times with 1 × PBS and incubated with fluorophore-conjugated secondary antibodies as required and supplemented with Hoechst stain (Sigma-Aldrich) for 1 h at room temperature in the dark. All secondary antibodies were diluted 1:2000 and Hoechst stain was diluted 1:4,000 in 3% BSA in 1 × PBS. Secondary antibodies used in this study were: Alexa Fluor 568 and Alexa Fluor 633 (Thermo Fisher Scientific). Samples were then washed 3 times with 1 × PBS and mounted using Prolong Gold mounting medium (Life Technologies). Confocal imaging was performed using the Olympus FV1200 Confocal.

### Golgi Disruption During *Salmonella* Infection

HeLa229 cells seeded onto coverslips were infected with derivatives of *Salmonella* SL1344. Samples were fixed with 4% paraformaldehyde in 1 × PBS for 10 min at room temperature at 20 h post infection and prepared for immunofluorescence analysis as described above. Primary antibodies used in this experiment were: polyclonal anti-Golgin 97 (Abcam, ab84830), and anti-*Salmonella* CSA-1 (BacTrace, 5310-0322); secondary antibodies used were: Alexa Fluor 488 and Alexa Fluor 568 (Thermo Fisher Scientific).

### Cytometric Bead Array Assay

RAW264.7 cells were infected with *S*. Typhimurium as above, with cell culture media replaced at 16 h post infection to focus on cytokine secretion at late stages of infection. Supernatants from RAW264.7 cells infected with various *Salmonella* strains were collected at 20 or 24 h post infection for analysis. Samples were processed for analysis using Cytometric Bead Array (CBA) Flex Sets (BD Biosciences) according to manufacturer's instructions. Samples were analyzed on the BD LSRFortessa^TM^ cell analyzer.

## Results

### SseK3 Glycosylates Rab1, Rab5, and Rab11 During *S*. Typhimurium Infection

**Figure 1:**
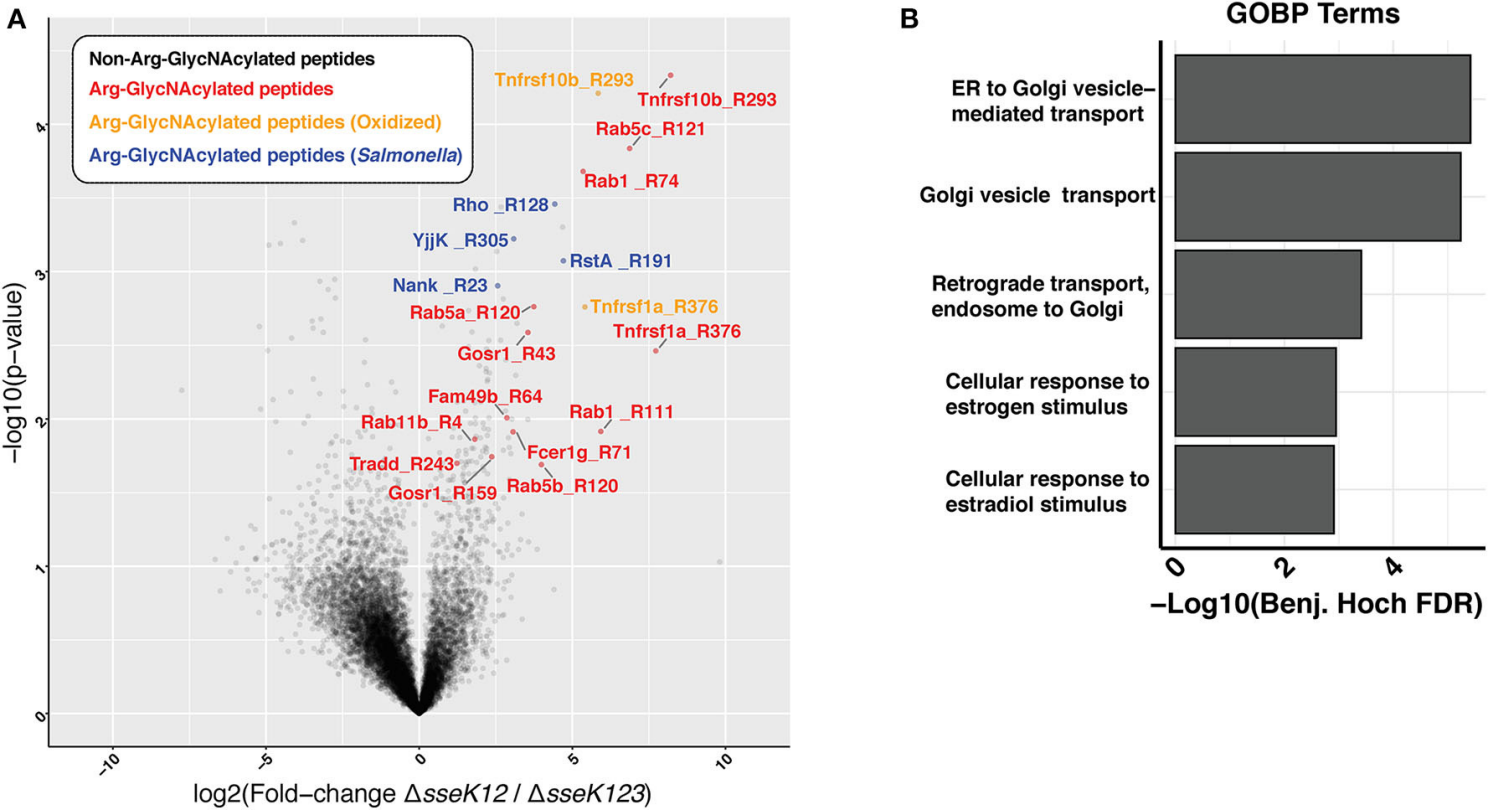
Enrichment of peptides Arg-GlcNAcylated by SseK3 derived from the insoluble fraction of *Salmonella*-infected RAW264.7 cell lysates. Arg-GlcNAcylated peptides were enriched and immunoprecipitated from the insoluble fraction of RAW264.7 cells infected with *S*. Typhimurium SL1344 Δ*sseK12* or *S*. Typhimurium SL1344 Δ*sseK123*. **(A)** The volcano plot depicts the mean ion intensity peptide ratios of Δ*sseK12* vs. Δ*sseK123* plotted against the -logarithmic student *t*-test *p*-values from biological triplicate experiments. Arg-GlcNAcylated peptides with a fold change > 2 and a *p* < 0.05 are highlighted. Peptides are labeled by their gene names followed by the location of the Arg-GlcNacylated arginine. Arg-GlcNAcylated peptides from the host cell are highlighted in red; Methionine oxidized Arg-GlcNAcylated peptides from host cell are highlighted in yellow; Arg-GlcNAcylated peptides from *Salmonella* are highlighted in blue. **(B)** Bar chart of enrichment analysis of GO-terms (Biological processes) associated with Arg-GlcNAcylated peptides compared to all observed peptides from immunoprecipitation experiments. Fisher's exact enrichment analysis demonstrates the over-representation of Golgi-associated processes with Arg-GlcNAcylated proteins in the insoluble membrane immunoprecipitation.

Using a mass spectrometry-based approach on total cell lysates, we previously identified TNFR1 and TRAILR as targets of SseK3 during *Salmonella* infection of RAW264.7 cells (Newson et al., 2019). However, given that SseK3 exhibits strong Golgi localization during *Salmonella* infection (Gunster et al., 2017), we postulated that SseK3 may also target Golgi-associated membrane proteins. To test this, we enriched membrane fractions from *Salmonella*-infected RAW264.7 cells at 20 h post infection, and digested these to produce peptides that were then immunoprecipitated using an antibody that recognizes Arg-GlcNAc and then analyzed by mass spectrometry. To identify the Arg-GlcNAcylated targets of SseK3 we analyzed the peptides immunoprecipitated from RAW264.7 cells infected with *S*. Typhimurium Δ*sseK12* and compared these to RAW264.7 cells infected with *S*. Typhimurium Δ*sseK123*. This approach revealed Arg-GlcNAcylation of previously identified SseK3 targets, TRAILR (Tnfrsf10b) and TNFR1 (Tnfrsf1a) (Newson et al., 2019), as well as novel host and bacterial protein Arg-GlcNAcylation events (Figure 1A, Supplementary Tables 1, 2). Consistent with the Golgi localization of SseK3, the Arg-GlcNAcylated targets identified were enriched for GO terms associated with Golgi-related biological processes including “ER to Golgi vesicle– mediated transport”, “Golgi vesicle transport” and “Retrograde transport, endosome to Golgi” (Figure 1B, Supplementary Table 3). Among the Golgi-related targets identified were several Rab GTPases, including Rab1, Rab5, and Rab11 (Figure 1A, Supplementary Tables 1, 2).

**Figure 2:**
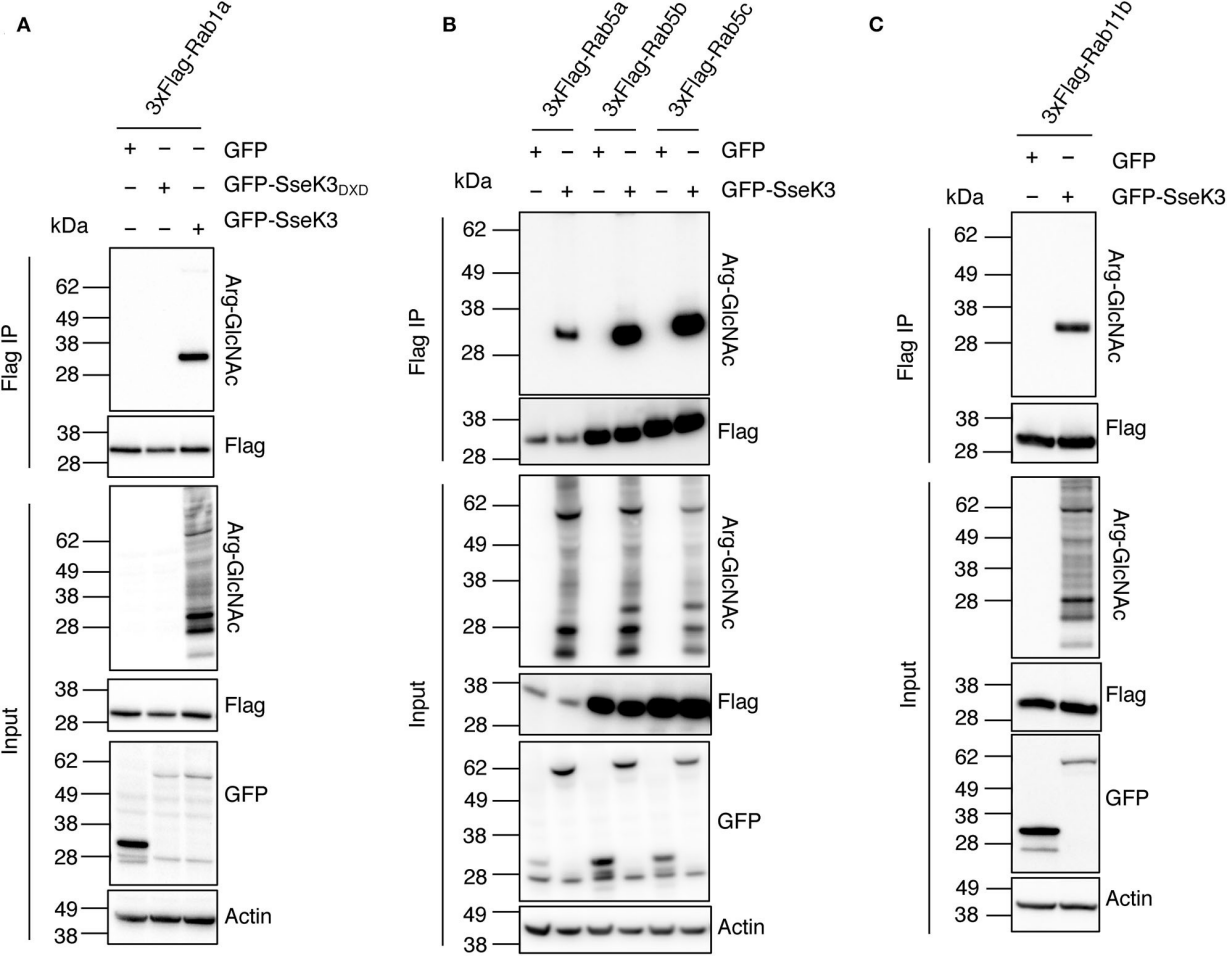
SseK3 modifies Rab1, Rab5, and Rab11 in co-transfected HEK293T cells. pEGFP-C2, pEGFP-C2-SseK3 or pEGFP-C2-SseK3_DXD_ were co-transfected with either p3xFlag-Rab1a **(A)**, p3xFlag-Rab5a/b/c **(B)**, or p3xFlag-Rab11b **(C)** into HEK293T cells before 3xFlag-tagged proteins were immunoprecipitated. Input and immunoprecipitate (IP) were subjected to immunoblot analysis with anti-ArgGlcNAc, anti-Flag M2-HRP, anti-GFP or anti-β-actin antibodies. Representative immunoblots of at least 3 independent experiments.

Rab GTPases are master regulators of intracellular vesicle transport. Rab5 and Rab11 are regulators of early endosomes and recycling endosomes respectively; while Rab1 mediates vesicle transport from the ER to Golgi and can be found on Golgi membranes (Zhen and Stenmark, 2015; Prashar et al., 2017). To confirm that Rabs are modified by SseK3, we co-transfected HEK293T cells with 3xFlag-tagged Rab1a, Rab5a/b/c or Rab11b and GFP tagged SseK3, and then performed anti-Flag immunoprecipitation and subsequent immunoblot analysis using Arg-GlcNAc antibodies. GFP or the catalytically inactive glycosyltransferase motif mutant GFP-SseK3_DXD_ were used as controls. We found that SseK3 does modify human Rab1a (Figure 2A), Rab5a/b/c (Figure 2B) and Rab11b (Figure 2C) with GlcNAc in transfected mammalian cells.

### Site-Directed Mutagenesis of Human Rab1 Confirms SseK3 Modifies Arg74, Arg82, and Arg111

**Figure 3:**
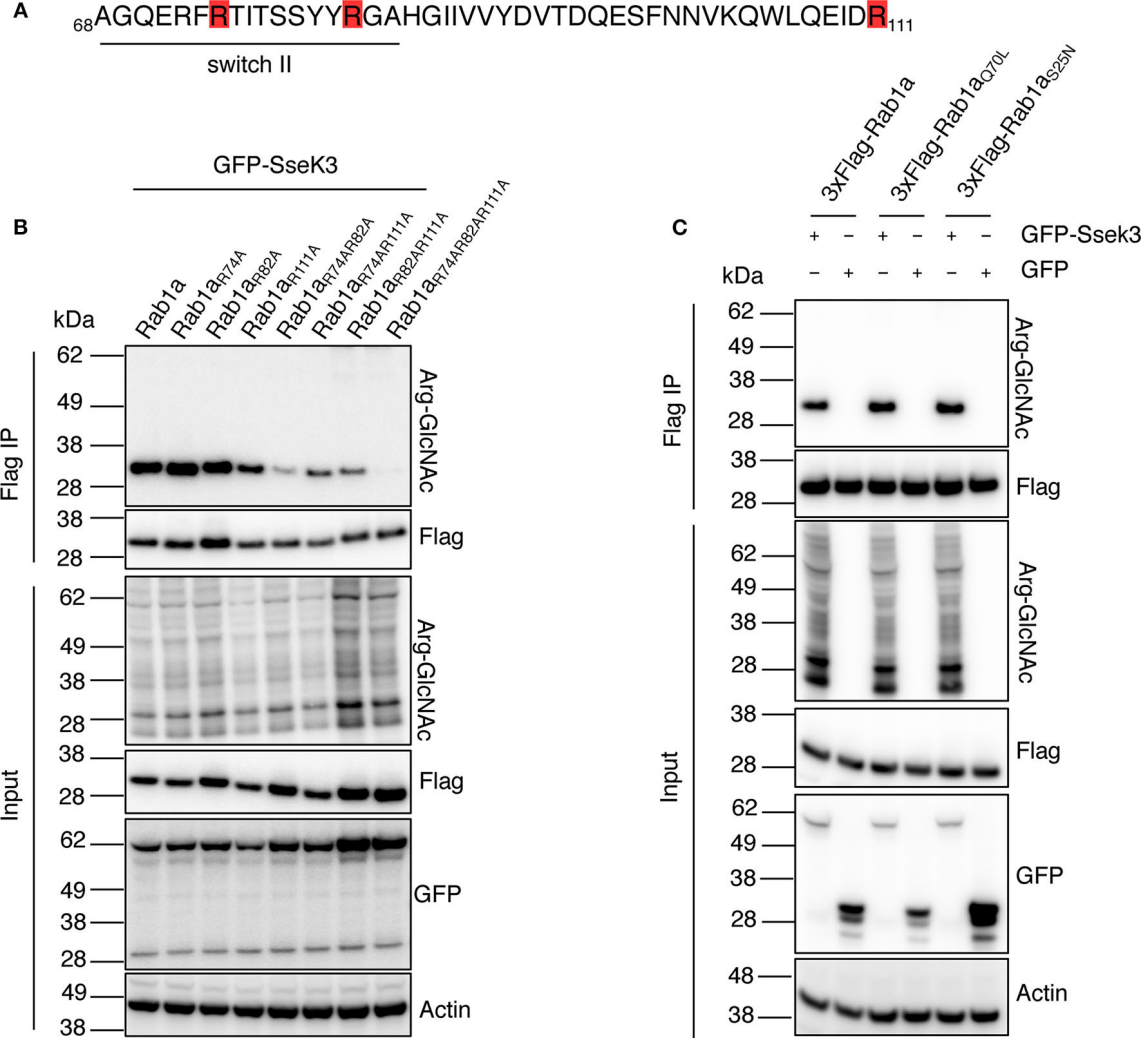
SseK3 modifies Arg74, Arg82, and Arg111 within Rab1a and has no preference for GTP-bound or GDP-bound Rab1a. **(A)** Amino acid sequence of Rab1a containing the arginine residues modified by SseK3 (highlighted in red) and the Rab1 switch II region (underlined). **(B)** GFP-SseK3 was co-expressed with 3xFlag tagged Rab1a arginine site-directed mutants in HEK293T cells. Flag-immunoprecipitation was performed on the cell lysates with subsequent immunoblot analysis using anti-ArgGlcNAc, anti-Flag M2-HRP, anti-GFP or anti-β-actin antibodies. Representative immunoblots of at least 3 independent experiments. **(C)** GFP or GFP-SseK3 together with 3xFlag tagged Rab1a or Rab1a nucleotide binding state mutants (active-state mimetic Rab1a_Q70L_, or constitutively inactive GDP-bound Rab1a_S25N_) were co-expressed in HEK293T cells by transfection. 3xFlag tagged proteins were immunoprecipitated for immunoblot analysis with anti-ArgGlcNAc, anti-Flag M2-HRP, anti-GFP or anti-β-actin antibodies. Representative immunoblots of at least 3 independent experiments.

Our mass spectrometry analysis revealed three different SseK3-mediated modification sites within Rab1 (corresponding to Arg74, Arg82, and Arg111 in Rab1a), whereas SseK3 modified single arginine residues in Rab5 and Rab11 respectively (Figure 1A, Supplementary Tables 1, 2). Strikingly, two of the Rab1 modification sites were located in the _74_RTITSSYYR_82_ peptide within the catalytic switch II region (Figure 3A). In contrast, the Rab5 and Rab11 modification sites were not located in this region, occurring on Arg120 in the third α-helix and Arg4 at the N-terminus respectively, and the roles of these residues in Rab activity are unknown. The switch II region of Rab1 is a hotspot for post-translational modifications by different bacterial effectors to regulate Rab1 activity (Muller et al., 2010; Mukherjee et al., 2011; Wang et al., 2018). The Rab switch II region, in addition to the switch I region is involved in nucleotide binding, and shift from being unfolded in the GDP-bound state to adopting well-defined conformations in the GTP-bound state to allow for Rab interactions with host effector proteins (Zhen and Stenmark, 2015). Given the importance of the switch II region, we focussed on Rab1a modification by SseK3. To confirm the SseK3 modification sites within Rab1a, we mutated the arginine residues of interest to alanines, which cannot be GlcNAcylated. Individual 3xFlag-tagged Rab1 mutants were co-expressed in HEK293T cells with GFP-tagged SseK3 and then subjected to Flag immunoprecipitation and immunoblot analysis. We found mutation of the arginine residues individually did not significantly impact Arg-GlcNAcylation of Rab1 by SseK3 (Figure 3B). Mutating two residues at a time had a modest effect on blocking modification and mutating all of Arg74, Arg82, and Arg111 resulted in complete abrogation of Rab1a Arg-GlcNAcylation, suggesting SseK3 modifies each of these three arginine residues (Figure 3B). Rab1a_R74AR82A_ was less efficiently modified by SseK3 compared to the other double site mutants, suggesting SseK3 may preferentially modify these two arginine residues located within the critical switch II region of Rab1a (Figure 3B). We then examined the intracellular localization of 3xFlag-tagged Rab1a arginine mutants by confocal microscopy. 3xFlag-tagged Rab1a or arginine mutants were expressed in HEK293T cells before the cells were stained with anti-golgin-97 antibodies for immunofluorescence analysis. 3xFlag-Rab1a showed a staining pattern consistent with Golgi localization (Supplementary Figure 1). All other mutants showed similar localization patterns, other than those containing a mutation of Arg82 (Supplementary Figure 1). All of 3xFlag-Rab1a_R82A_, 3xFlag-Rab1a_R74AR82A_, 3xFlag-Rab1a_R82AR111A_ and 3xFlag-Rab1a_R74AR82AR111A_ were instead expressed throughout the cell (Supplementary Figure 1).

### SseK3 Modifies Both GTP-Bound and GDP-Bound Rab1

Rab GTPases cycle between a GTP-bound active state and GDP-bound inactive state to mediate different steps of vesicle trafficking. The Rab switch regions undergo major conformational changes depending on the nucleotide binding state (Pfeffer, 2005). These changes may influence the ability of effectors to bind and modify the Rabs. For example, the *Legionella* glucosyltransferase effector, SetA preferentially modifies GDP-bound Rab1 (Wang et al., 2018), and an endogenous Rab1 regulator, TAK1, also preferentially phosphorylates the GDP-bound form of Rab1 (Levin et al., 2016). Thus, we explored whether SseK3 exhibited a preference for GTP-bound or GDP-bound Rab1a. A constitutively inactive 3xFlag-tagged GDP-bound Rab1a_S25N_ (Nuoffer et al., 1994) or active-state mimetic Rab1a_Q70L_ (Tisdale et al., 1992) were co-transfected with GFP-SseK3 into HEK293T cells before being subjected to Flag-immunoprecipitation and immunoblot analysis. No significant difference in Arg-GlcNAc modification was observed among Rab1a, Rab1a_S25N_ or Rab1a_Q70L_ (Figure 3C). Thus, modification of Rab1a by SseK3 was independent of the Rab1a nucleotide binding state. In this way, SseK3 functions differently in comparison to the endogenous Rab1 regulator TAK1, or the glucosyltransferase bacterial effector, SetA.

### SseK2 and SseK3 Co-localize With Rab1 at the Golgi

**Figure 4:**
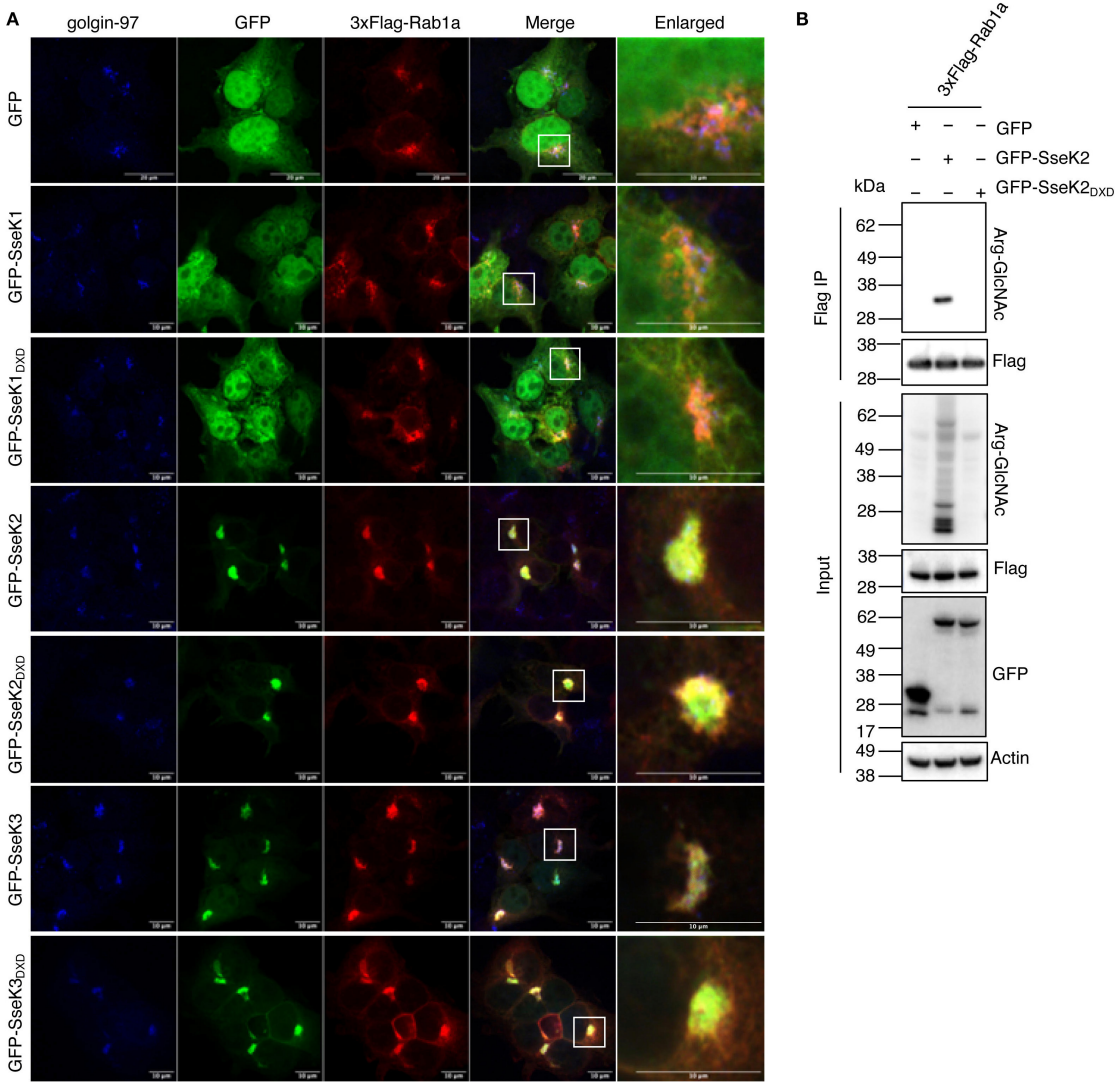
SseK2 modifies Rab1a and both SseK2 and SseK3 co-localize with Rab1a at the Golgi. **(A)** The intracellular localization pattern of 3xFlag-Rab1a was examined in the presence of GFP, GFP-SseK1, GFP-SseK2, GFP-SseK3 and their catalytically inactive mutants by confocal microscopy in transfected HEK293T cells. Anti-golgin-97 and anti-Flag antibodies were used to identify the Golgi and recombinant 3xFlag-Rab1a respectively. White boxes highlight Golgi that are shown enlarged. Representative immunofluorescence fields of at least 3 independent experiments **(B)** SseK2 also modifies Rab1a with Arg-GlcNAc. pEGFP-C2, pEGFP-C2-SseK2 or pEGFP-C2-SseK2_DXD_ were co-transfected with p3xFlag-Rab1a into HEK293T cells before 3xFlag-tagged proteins were immunoprecipitated. Input and immunoprecipitate (IP) were subjected to immunoblot analysis with anti-ArgGlcNAc, anti-Flag M2-HRP, anti-GFP or anti-β-actin antibodies.

We next investigated the cellular localization of Rab1a in the presence of the SseKs. GFP, GFP-SseK1, GFP-SseK2, GFP-SseK3 or their catalytically inactive mutants were co-expressed with 3xFlag-tagged Rab1 in HEK293T cells before the cells were stained with anti-golgin-97 antibodies for immunofluorescence analysis. 3xFlag-Rab1a showed a Golgi-associated staining pattern in all samples tested, while both GFP and GFP-SseK1 were expressed throughout the cell (Figure 4A). Consistent with previous findings, GFP-SseK3 also localized to the Golgi in HEK293T cells (Figure 4A) (Gunster et al., 2017). Furthermore, we observed that GFP-SseK3_DXD_ also localized to the Golgi with 3xFlag-Rab1a, indicating localization was independent of Arg-GlcNAcylation activity (Figure 4A).

SseK2 also localizes to the Golgi during *Salmonella* infection (Gunster et al., 2017), and may therefore share targets with SseK3. We found that SseK2 and Rab1a also co-localized at the Golgi, and this was also independent of SseK2 catalytic activity (Figure 4A). This suggested that SseK2 may also target Rab1a for Arg-GlcNAcylation. Flag-immunoprecipitation and immunoblot analysis confirmed Arg-GlcNAcylation of 3xFlag-Rab1a by GFP-SseK2 in co-transfected HEK293T cells (Figure 4B).

Inhibition of Rab1 can lead to disruption of the Golgi (Dong et al., 2012). In our co-transfection studies we did not observe Golgi disruption (Figure 4A). However, we also examined the Golgi in cells that were not overexpressing Rab1, and found that after 20 h of infection with wild type *S*. Typhimurium SL1344, 15% of infected cells showed Golgi disruption, but this was independent of SseK2 or SseK3 (Supplementary Figure 2).

### SseK3 Inhibits Host Protein Secretion During Transfection and *S*. Typhimurium Infection

**Figure 5:**
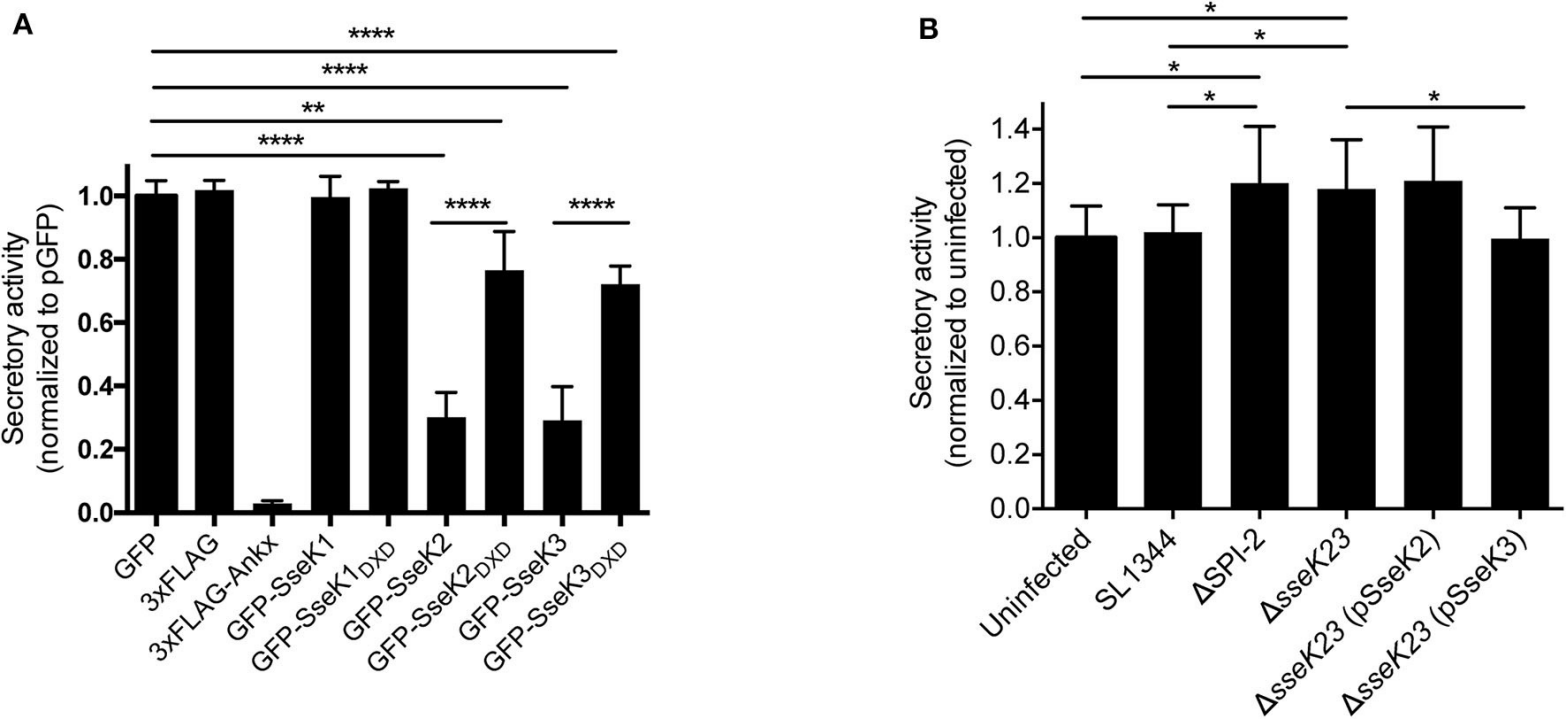
SseK3 inhibits the secretion of alkaline phosphatase in transfected HEK293T cells and during *Salmonella* infection. **(A)** pSEAP was co-transfected with mammalian expression vectors expressing GFP, 3xFlag, 3xFlag-AnkX, GFP-SseK1, GFP-SseK2, GFP-SseK3 or their catalytic mutants into HEK293T cells. Supernatants and cell lysates were then analyzed for alkaline phosphatase activity. Secretory activity was calculated as alkaline phosphatase activity in the supernatants divided by total alkaline phosphatase activity, which includes alkaline phosphatase activity in both supernatants and cell lysates. These were then normalized to the GFP expressing sample. Results are mean + SD of three independent experiments performed in duplicate. ****P < 0.0001, **P < 0.01; unpaired, two-tailed *t*-test. **(B)** HeLa229 cells stably expressing SEAP were infected with various *Salmonella* SL1344 strains. The cell culture media was replaced at 16 h post infection, and infection allowed to proceed for a further 8 h. Supernatants and cell lysates were analyzed for alkaline phosphatase activity at 24 h of infection. Secretory activity was calculated as alkaline phosphatase activity in the supernatants divided by total alkaline phosphatase activity, normalized to uninfected cells. Results are mean + SD of three independent experiments performed in duplicate. *P < 0.05; unpaired, two-tailed *t*-test.

As Rab1 mediates vesicle transport from the ER to Golgi early in the secretory pathway, we explored the functional consequences of Arg-GlcNAcylation on Rab1 activity by testing whether the SseK family of proteins inhibited host protein secretion. We employed secreted embryonic alkaline phosphatase (SEAP) as a reporter to examine the activity of the secretory pathway in transfected cells. AnkX, a *Legionella* effector which significantly inhibits SEAP secretion (Mukherjee et al., 2011), was adopted as a positive control for the assay. Vectors expressing 3xFlag, 3xFlag-AnkX, GFP, GFP-SseK1, GFP-SseK2, GFP-SseK3 or their catalytically inactive DXD motif mutants were co-transfected into HEK293T cells with a SEAP expressing vector. As expected, expression of 3xFlag-AnkX significantly inhibited the secretion of SEAP in comparison to cells expressing 3xFlag only (Figure 5A). Expression of either GFP-SseK2 or GFP-SseK3 significantly inhibited the secretion of SEAP compared to GFP or GFP-SseK1 expressing cells (Figure 5A). Catalytically inactive SseK2 and SseK3 also partially inhibited the secretion of SEAP; however, this was not to the level of inhibition mediated by active SseK2 and SseK3 (Figure 5A).

We next explored whether SseK2 and SseK3 inhibited host protein secretion during *S*. Typhimurium SL1344 infection. A SEAP expressing HeLa229 reporter cell line was constructed and infected with derivatives of *S*. Typhimurium before a SEAP assay was performed. To analyse protein secretion at a timepoint when SseK2 and SseK3 are likely to be active, the cell media was changed at 16 h post infection, and infection allowed to proceed for a further 8 h before SEAP analysis at 24 h post infection. Wild type *S*. Typhimurium SL1344-infected cells showed similar levels of SEAP secretion compared to uninfected cells. Complex manipulation of host signaling pathways occurs during infection, thus SL1344 infection may simultaneously activate and interfere with host cell protein secretion. In support of this, modest increases in SEAP secretion were observed from cells infected with either ΔSPI-2 or Δ*sseK23 Salmonella* strains compared to cells infected with *S*. Typhimurium SL1344 or uninfected cells (Figure 5B). However, SEAP secretion returned to the levels observed for SL1344-infected cells only upon complementation of Δ*sseK23* with SseK3, and not SseK2 when over-expressed from a plasmid (Figure 5B). Hence, although both SseK2 and SseK3 robustly inhibited the secretion of SEAP when transfected into cells, we were unable to confirm a role for SseK2 in inhibition of the host cell secretory pathway in the context of infection, and SseK3 had only a modest impact on SEAP secretion during *Salmonella* infection. To control for bacterial numbers in these experiments, we examined intracellular replication of *S*. Typhimurium SL1344 and its derivatives and found that compared to wild type SL1344, only the SPI-2 mutant showed significantly impaired replication in the HeLa229 SEAP cell line (Supplementary Figure 3A).

### Effect of SseK3 on Cytokine Secretion During Infection of RAW264.7 Cells

**Figure 6:**
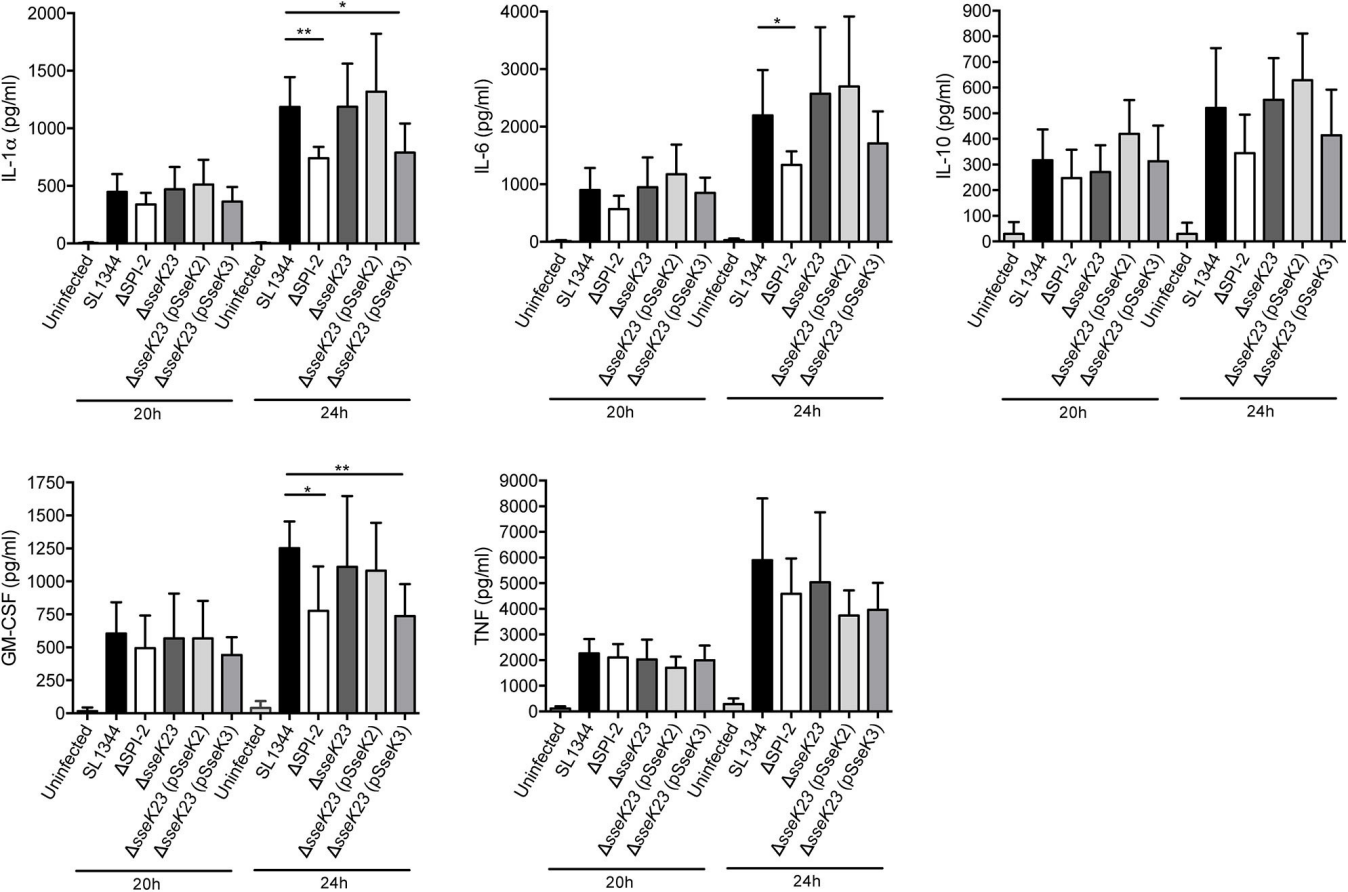
SseK3 does not inhibit the secretion of cytokines during *Salmonella* infection of RAW264.7 cells. Supernatants from RAW264.7 cells infected with wild type, ΔSPI-2, Δ*sseK23*, Δ*sseK23* (pSseK2), or Δ*sseK23* (pSseK3) *S*. Typhimurium SL1344 strains were collected for cytometric bead array analysis. After 16 h of infection, the cell culture media was changed, and supernatants were collected at 20 or 24 h post infection. Concentrations of cytokines in supernatants were determined based on fluorescence intensities from PE conjugated beads by flow cytometry compared to a standard curve and represented as pg/ml. Results are mean + SD of three independent experiments performed in duplicate. *P < 0.05, **P < 0.01; unpaired, two-tailed *t*-test.

Given their potential effect on host cell protein export, we next explored whether SseK2 or SseK3 inhibited cytokine secretion during *Salmonella* infection. RAW264.7 cells were infected with wild type *S*. Typhimurium SL1344 or mutant derivatives for 16 h before the cell culture media was changed and then supernatants were collected and analyzed for cytokine levels at 20 and 24 h post infection using a cytometric bead array. Replication of the strains at 24 h post infection was also examined, with only the SPI-2 mutant showing impaired replication in RAW264.7 cells (Supplementary Figure 3B). Compared to uninfected cells, *S*. Typhimurium SL1344 infection resulted in increased cytokine secretion at both 20 and 24 h post infection (Figure 6). In contrast to the SEAP assay, ΔSPI-2 infection of RAW264.7 cells resulted in less IL-1α, IL-6 and GM-CSF cytokine secretion compared to wild type-infected samples at 24 h of infection and no significant differences were observed between wild type SL1344 and Δ*sseK23* infected cells (Figure 6). A reduction in IL-1α and GM-CSF secretion was observed when SseK3 was over-expressed in the Δ*sseK23* mutant compared to SL1344-infected cells, but not when compared to Δ*sseK23*-infected cells (Figure 6). In summary, during infection SseK3 appeared to have a greater effect on Rab1-dependent host protein secretion than SseK2, but overall SseK3 had only a marginal influence on the secretion levels of some cytokines when overexpressed.

## Discussion

Rab GTPases are well known for their role in mediating endocytosis and exocytosis as well as other intracellular membrane trafficking events. Many bacterial pathogens including *Salmonella* hijack Rab-dependent pathways to facilitate infection (Spano and Galan, 2018). For example, maturation of the SCV requires participation of several key intracellular vesicle transport regulators, including Rab5, Rab7, and Rab11 (Knodler and Steele-Mortimer, 2003; Brumell and Grinstein, 2004; Smith et al., 2005). Upon invasion, *Salmonella* modulates Rab recruitment to the SCV in a SPI-1-dependent manner (Smith et al., 2007). Rab5 is recruited to the SCV by the SPI-1 effector SopB, which is a phosphoinositide phosphatase (Mallo et al., 2008), while another SPI-1 effector, SopE, functions as a guanine exchange factor (GEF) for Rab5 and promotes the formation of GTP-bound active Rab5 on the SCV (Mukherjee et al., 2001). The enhanced retention of active Rab5 on the SCV is hypothesiszed to promote fusion with early endosomes and prevent trafficking to mature lysosomes (Parashuraman and Mukhopadhyay, 2005; Madan et al., 2008). Rab11 is also recruited to early SCVs following *Salmonella* invasion, and is involved in SCV maturation but is not essential for replication (Smith et al., 2005, 2007). Many studies of Rab manipulation by *Salmonella* focus on early time points of infection before the complete repertoire of SPI-2 effectors are expressed and translocated into host cells. Less is known about the impact of *Salmonella* on the host endosomal pathway at later stages of infection, although two SPI-2 effectors, SopD2 and GtgE are known to target Rabs including Rab32. SopD2 functions as a GTPase activating protein (GAP) for Rab32 (Spano et al., 2016) while GtgE is a cysteine protease that cleaves Rab32 (Spano and Galan, 2012, 2018; Wachtel et al., 2018). Together, these effectors inhibit recruitment of Rab32 to the SCV and subsequent Rab32-mediated control of replication (Spano and Galan, 2012; Spano et al., 2016).

In this study, we identified Rab1, Rab5, and Rab11 as host targets of the SPI-2 effector, SseK3 during *Salmonella* infection. These targets were identified by mass spectrometry and confirmed by immunoblot of ectopically expressed Rabs immunoprecipitated from HEK293T cells co-expressing SseK3. Using a SEAP reporter assay we found SseK3 impaired host protein secretion in transfected cells and modestly reduced secretion levels during *Salmonella* infection, suggesting Arg-GlcNAcylation of Rab1 by SseK3 at least partially blocked Rab1 activity. However, SseK3 did not appear to reduce the secretion of selected cytokines during *Salmonella* infection when expressed and translocated at native levels. Interestingly, inactive SseK3_DXD_ and SseK2_DXD_ also partially inhibited SEAP secretion in transfected cells, but the reason for this is unclear.

While this study was under review, another group also reported that Rab1 is Arg-GlcNAcylated by SseK3 (Meng et al., 2020). In contrast to our work, Meng et al. concluded that SseK3 did inhibit cytokine secretion during infection, while the impact of SseK2 on Rab1 and host protein secretion was not examined (Meng et al., 2020). Whereas Meng et al. performed infections with a triple mutant of *S*. Typhimurium SL1344 lacking all of *sseK1, sseK2* and *sseK3*, we used a double *sseK2/sseK3* mutant for our studies. Given that the strains we used retained SseK1, which together with SseK3 can inhibit inflammatory signaling through TNFR1 (Gunster et al., 2017; Newson et al., 2019), differences in the results observed by Meng et al. compared to our study could be due to altered cytokine expression levels due to the presence or absence of SseK1 (Meng et al., 2020). Thus, the SEAP reporter was a more direct measure of the ability of SseK3 to block the host cell secretory pathway as its expression was not influenced by inflammatory signaling. Although a previous study aimed at discovering *Salmonella* effector proteins that interact with host exocytic pathway failed to identify SseK2 and SseK3 as inhibitors of SEAP secretion, (Perrett and Zhou, 2013), this may have been due to insufficient expression of SseK2 and SseK3, which was not determined.

Two Rab1-targeting SPI-2 effectors, SseF and SseG, were recently reported to inhibit host autophagy during infection by abolishing Rab1 activation, indicating Rab1 is targeted by *Salmonella* once the SCV is established (Feng et al., 2018). It is not surprising that *Salmonella* employs multiple effectors to modulate different Rab1-related host cell events, considering intracellular pathogens need to counteract multiple host cell defense pathways. Previous studies on *Legionella* provide a good example of how intracellular pathogens utilize different effectors to modulate Rab1, even with contradictory impacts. During *Legionella* infection, spatio-temporal regulation of Rab1 activity is achieved largely via several post-translational modifications. For example the *Legionella* Dot/Icm effector SidM/DrrA AMPylates Tyr_80_ of Rab1 to retain it in the GTP-bound active state; while this post-translational modification is reversed by another effector, SidD (Muller et al., 2010; Mukherjee et al., 2011; Tan and Luo, 2011). Another *Legionella* effector, AnkX modifies Ser_79_ with a phosphocholine moiety and this modification is eliminated by a dephosphorylcholinase effector, Lem3 (Tan et al., 2011). Furthermore, a *Legionella* glucosyltransferase effector SetA modifies Thr_75_ of Rab1 with a glucose molecule and thus limits GTPase activity (Wang et al., 2018). Interestingly, most sites on Rab1 targeted for post-translational modification by different bacterial effectors, as well as the endogenous Rab1 regulator TAK1 (Levin et al., 2016), are located within the _74_RTITSSYYR_82_ peptide of the switch II region, highlighting the susceptibility of this region to attack and/or regulation. Notably, we found SseK3 modified three different arginine residues in Rab1a, Arg74, Arg82 and Arg111, two of which were located within the Rab1 switch II region. Meng et al. identified the same SseK3-mediated Arg-GlcNAc modification sites within Rab1, and an additional modification at Arg72 when SseK3 was expressed from a multi-copy plasmid during *S*. Typhimurium infection (Meng et al., 2020).

We did not directly examine the activity of Arg-GlcNAc modified Rab1 in this study, however Meng et al. reported that SseK3-modified Rab1 had reduced GTPase activity, impaired interaction with binding partners and the membrane cycling of Rab1 was also perturbed (Meng et al., 2020). Our observations that SseK3 modified Rab1 regardless of its nucleotide binding state was also supported by Meng et al. (2020) and suggests that Arg-GlcNAc modified Rabs may also be present in the soluble fraction of *S*. Typhimurium-infected cells, as GDP-bound Rabs are not membrane bound (Zhen and Stenmark, 2015; Prashar et al., 2017). Indeed, we previously identified Arg-GlcNAc modified peptides from Rab1 and Rab5 in total cell lysates from RAW246.7 cells infected with *S*. Typhimurium Δ*sseK12*, although the modified Rab peptides were either not detected in all three biological replicates or were detected at lower levels compared to TNFR1 and TRAILR2 (Newson et al., 2019).

Similar to Meng et al., we found that SseK3 and SseK2 exhibited Golgi-associated localization with Rab1a, but that this was independent of Arg-GlcNAc activity. SseK3 localized to the *cis-*Golgi independently of its catalytic motif, with localization mediated by a polybasic region within SseK3 that binds phospholipids (Meng et al., 2020). However, whereas Meng *et al*. reported that ectopic expression of SseK3 caused fragmentation of the Golgi (Meng et al., 2020), we did not observe altered Golgi morphology when either SseK2 or SseK3 were translocated at native levels during *S*. Typhimurium infection. Thus the observations by Meng *et al*. could be artifacts of SseK3 over-expression, making the physiological relevance of SseK3-mediated Golgi disruption during infection unclear (Meng et al., 2020). This may also suggest that Rab1 is not a primary target of SseK3 during infection, or that levels of non-modified Rab1 are still sufficient to maintain Golgi structure. Notably, no host or pathogen factors have been reported to reverse Arg-GlcNAcylation (Scott et al., 2017). As such, the impact of Arg-GlcNAc modification on intracellular vesicle transport could still be significant over time even when only a small portion of target protein is modified. Further work is required to examine the effect of SseK3 on Rab5 or Rab11 function, as they may be preferred targets of SseK3 during *S*. Typhimurium infection. Interestingly, Meng *et al*. did not report Rab5 or Rab11 as significant targets of SseK3 during infection. Discrepancies between the targets of SseK3 identified may be due to the different approaches and cell lines used. Our Arg-GlcNAc pulldowns were performed on insoluble fractions of infected RAW246.7 cells, while Meng *et al*. identified modified proteins in cell lysates from transfected or infected HEK293T cells, or infected HeLa, iBMDM or MEF cells (Meng et al., 2020).

In addition to Rab1, Rab5, and Rab11, we identified several *Salmonella* proteins that were Arg-GlcNAcylated by SseK3 during infection, including the transcription termination factor Rho and a two-component regulatory system factor, RstA. The consequences of these modifications have not been investigated here, however it is worthwhile noting that NleB of enterohaemorrhagic and enteropathogenic *E. coli* and *C. rodentium* also have intra-bacterial Arg-GlcNAcylation activity, and this impacts bacterial survival in oxidative stress conditions (El Qaidi et al., 2020). NleB1, SseK1, and SseK3 also perform auto-Arg-GlcNAcylation, and this is required for activity against death domain protein targets (Xue et al., 2020).

In summary, we found that the *Salmonella* glycosyltransferase effector SseK3 modified Rab1, Rab5, and Rab11 during *Salmonella* infection. SseK3 targeted critical arginine residues in the switch II region of Rab1, thereby influencing host cell protein secretion during infection. Hence, in addition to its role in blocking death receptor signaling (El Qaidi et al., 2017; Gunster et al., 2017; Newson et al., 2019), SseK3 may modify the host cell secretome during the later stages of *Salmonella* infection.

## Data Availability Statement

The datasets presented in this study can be found in online repositories. The names of the repository/repositories and accession number(s) can be found in the article/Supplementary Material.

## Author Contributions

JG designed and performed experiments and wrote the manuscript. NS and JN designed and performed mass spectrometry experiments. RW, TW, and GP made reagents and GN, ID, and JP provided experimental advice. CG and EH conceptualized and supervised the study and wrote the manuscript. All authors provided critical feedback and edited the manuscript.

## Conflict of Interest

The authors declare that the research was conducted in the absence of any commercial or financial relationships that could be construed as a potential conflict of interest.
