# Supplementary material for: The Salmonella Effector SseK3 Targets Small Rab GTPases
Source: Front Cell Infect Microbiol. 2020 Aug 19;10:419. doi: 10.3389/fcimb.2020.00419 (PMC7466453; doi:10.3389/fcimb.2020.00419)
Supplement: Supplementary file 1 [file Data_Sheet_1.pdf]

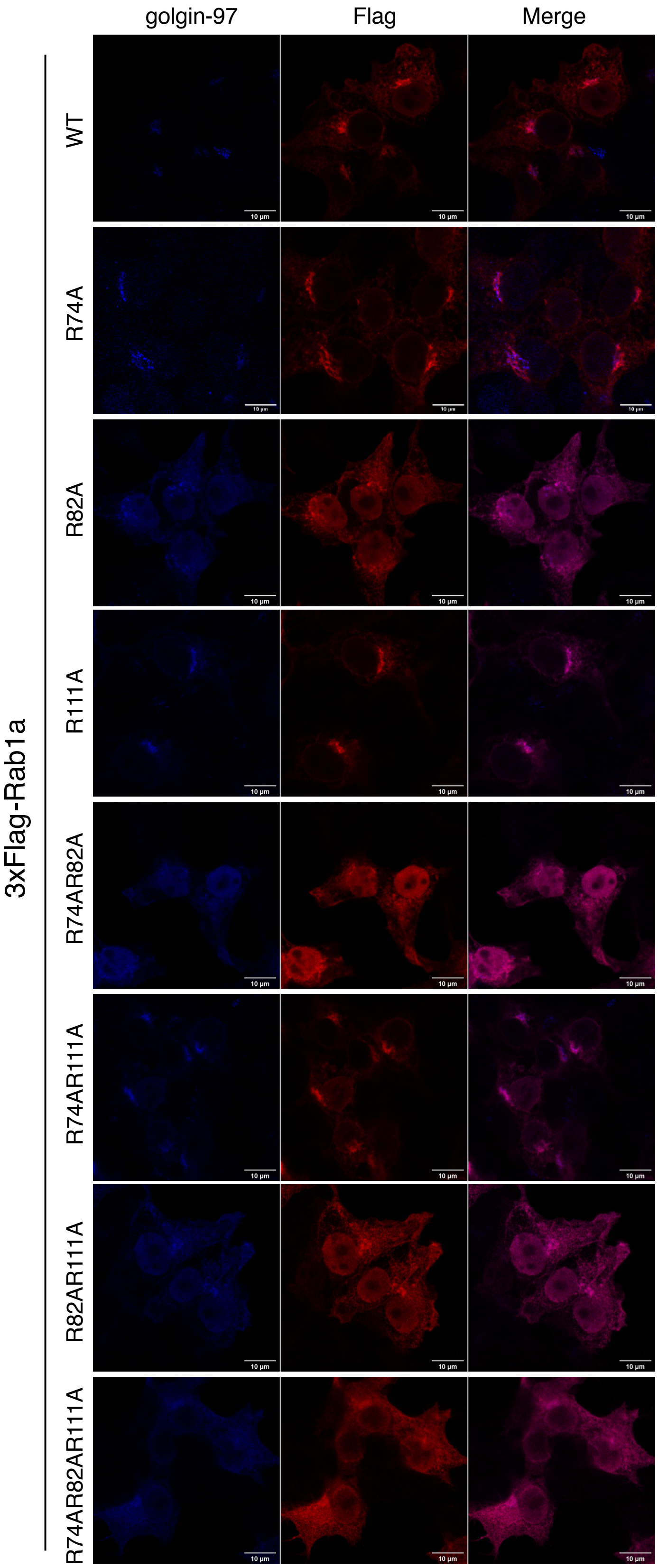

Supplementary Figure 1. Localisation of Rab1a arginine site-directed mutants.
The intracellular localisation pattern of wild type (WT) 3xFlag-Rab1a or arginine mutants were examined by confocal microscopy in transfected HEK293T cells. Anti-golgin-97 and anti-Flag antibodies were used to identify the Golgi and recombinant 3xFlag-Rab1a respectively. Representative immunofluorescence fields of 3 independent experiments.

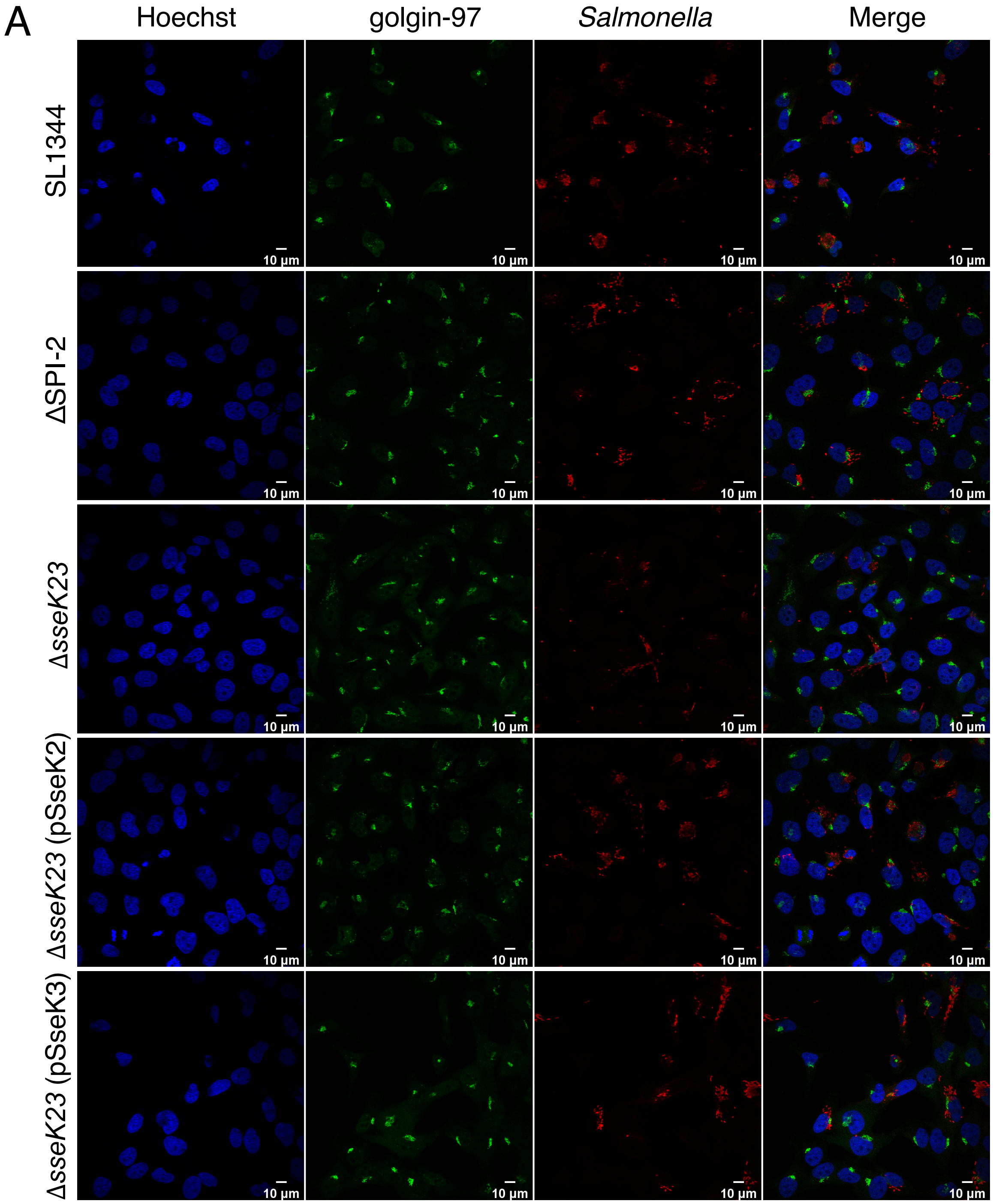
A
Hoechst
golgin-97
Salmonella
Merge
SL1344
ΔSPI-2
ΔsseK23
ΔsseK23 (pSseK2)
ΔsseK23 (pSseK3)
10 µm

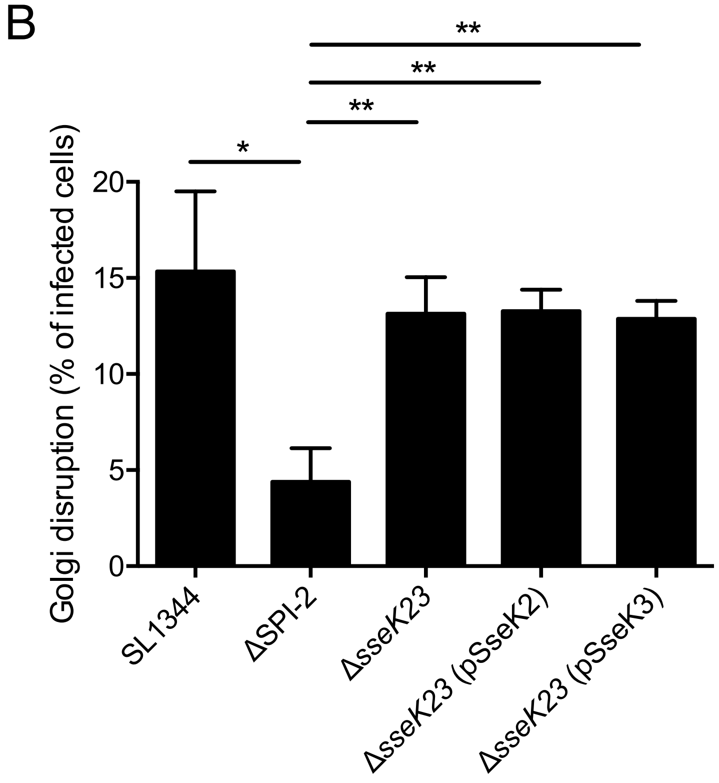

Supplementary Figure 2. Golgi disruption in *S.* Typhimurium-infected HeLa229 cells.
(A) Immunofluorescence microscopy showing HeLa229 cells infected with derivatives of *S.* Typhimurium SL1344 at 20 hours post infection. Cells are stained with Hoechst (blue), anti-golgin-97 or anti-*Salmonella* CSA-1. Representative immunofluorescence fields of at least 3 independent experiments.
(B) Quantification of infected cells with disrupted golgin-97 staining after 20 hours of *S.* Typhimurium SL1344 infection. Results are mean + SD of at least 100 cells counted for each of three independent experiments. *P < 0.05, **P < 0.01; unpaired, two-tailed t-test.

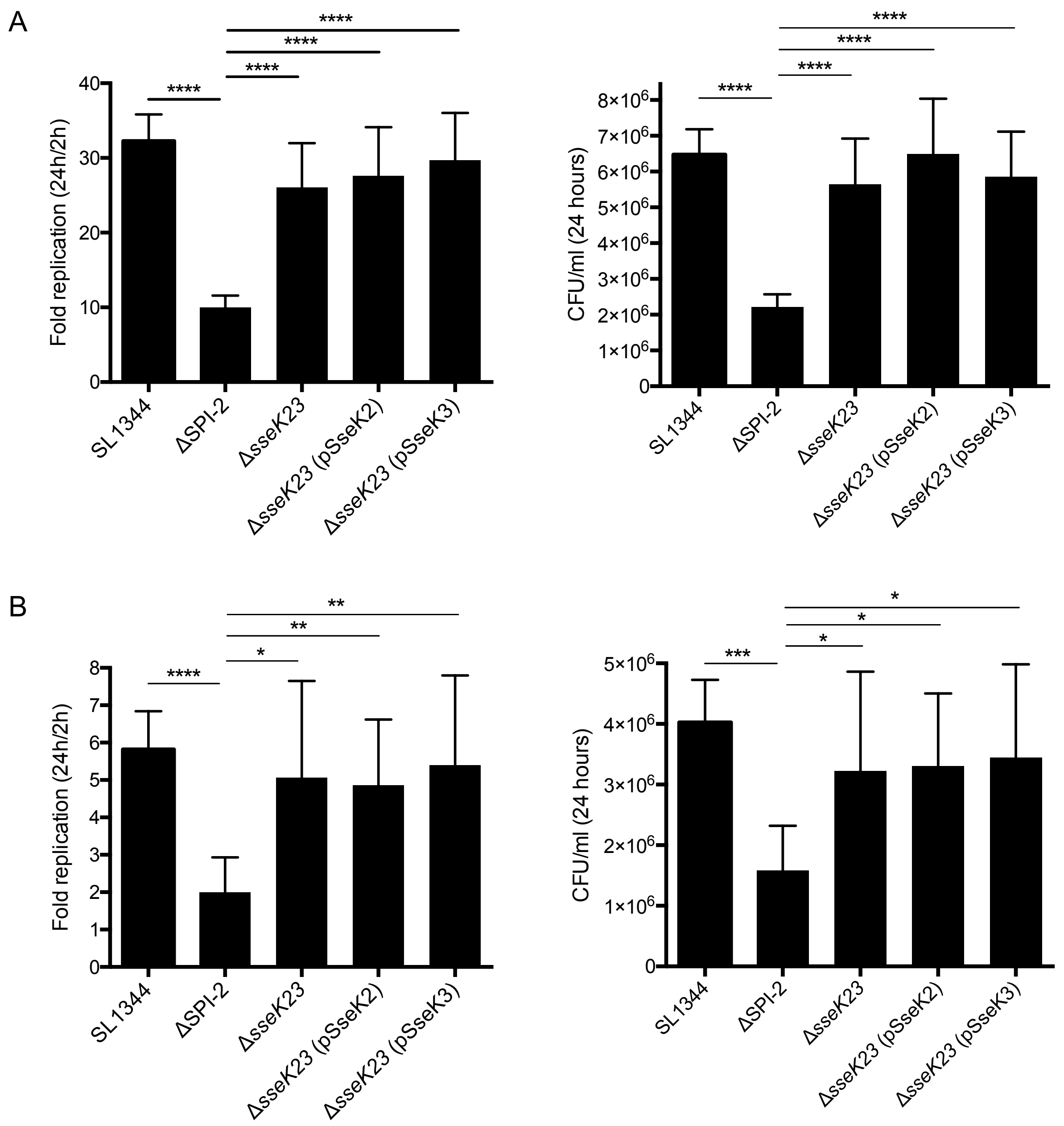

Supplementary Figure 3. Intracellular replication of the *Salmonella* SL1344 strains used in this study.

(A) Intracellular replication of *Salmonella* SL1344 derivatives in HeLa229 SEAP cells. Replication is expressed as a fold change in colony-forming units (CFU) after 24 hours compared to CFU after 2 hours of infection, or CFU/ml at 24 hours post infection. Results are mean + SD of three independent experiments performed in duplicate. ****P < 0.0001; unpaired, two-tailed t-test.

(B) Intracellular replication of *Salmonella* SL1344 derivatives in RAW 264.7 cells. Replication is expressed as a fold change in colony-forming units (CFU) after 24 hours compared to CFU after 2 hours of infection or CFU/ml at 24 hours post infection. Results are mean + SD of three independent experiments performed in duplicate. *P < 0.05, **P < 0.01, ***P < 0.001, ****P < 0.0001; unpaired, two-tailed t-test.
